# High-Dimensional Protein Analysis Uncovers Distinct Immunologic and Stromal Features in Primary and Metastatic Pancreatic Ductal Adenocarcinoma

**DOI:** 10.1158/0008-5472.CAN-25-1697

**Published:** 2025-12-19

**Authors:** Emily Greene, Natalie K. Horvat, Deon Bryant Doxie, Vaunita Cohen Parihar, Jayden Kim, Cameron J. Herting, Erin E. Grundy, Ayana T. Ruffin, Alyssa M. Krasinskas, Shishir K. Maithel, Juan M. Sarmiento, Mihir M. Shah, Mohammad Y. Zaidi, Maria Diab, Olatunji B. Alese, Kavita Dhodapkar, Haydn T. Kissick, Bassel F. El-Rayes, Chrystal M. Paulos, Gregory B. Lesinski

**Affiliations:** 1 Department of Hematology and Medical Oncology, Emory University, Atlanta, Georgia.; 2 Winship Cancer Institute of Emory University, Atlanta, Georgia.; 3 Department of Surgery, Emory University, Atlanta, Georgia.; 4 Department of Microbiology and Immunology, Emory University, Atlanta, Georgia.; 5 Department of Pathology, Emory University, Atlanta, Georgia.; 6 Division of Surgical Oncology, Northwestern University Feinberg School of Medicine, Chicago, Illinois.; 7 Department of Internal Medicine, Henry Ford Hospital, Detroit, Michigan.; 8 Department of Internal Medicine, Michigan State University, East Lansing, Michigan.; 9 Department of Pediatrics, Emory University, Atlanta, Georgia.; 10 Department of Urology, Emory University, Atlanta, Georgia.; 11 Department of Medicine, University of Alabama at Birmingham, Birmingham, Alabama.

## Abstract

Pancreatic ductal adenocarcinoma (PDAC) is an aggressive tumor type with poor patient outcomes. Most patients present with metastatic disease, which generally has reduced immune infiltration compared with primary tumors. Further work to elucidate the specific cellular features of metastatic PDAC is needed to guide the development of future immunotherapy strategies. In this study, we investigated the hypothesis that PDAC tumors harbor distinct immunologic and stromal features depending on their anatomic site. Multiplex IHC, spatial analysis, and single-cell mass cytometry (CyTOF) uncovered dominant immune and stromal cell populations in tumors derived from 27 primary and 26 liver metastases. Metastatic liver tumors from patients with PDAC contained reduced T-cell infiltration, fibroblast populations, and collagen accumulation than primary lesions, whereas CD68^+^ cells, often coexpressing CCR2, were more abundant. Spatial analyses revealed distinct immune cell communities in primary and metastatic PDAC, in which cytokeratin 19 (CK19^+^) cells clustered differentially with αSMA^+^, CD3^+^, and CD68^+^ cells, depending on the tumor site. When comparing tumor-associated regions, the proportion of peritumoral CK19^−^ cells remained consistent, but their composition varied by disease site. CD8^+^ T cells were significantly less frequent in metastatic tumors, whereas both CD4^+^ and CD8^+^ T cells present in primary tumors expressed more transcription factors associated with suppressive properties, including FoxP3 and RORγt. CyTOF revealed that T cells coexpressed multiple inhibitory checkpoint receptors, most notably LAG3 and PD-1. This report reveals that primary and metastatic tumors from patients with PDAC harbor vastly distinct immunologic and stromal features at the protein level.

**Significance:**

Protein level analysis reveals distinct immunological and stromal features between primary and metastatic pancreatic tumors, offering a rationale for immunotherapies that target macrophages and increase T-cell abundance in metastatic disease.

## Graphical Abstract

**Figure:**
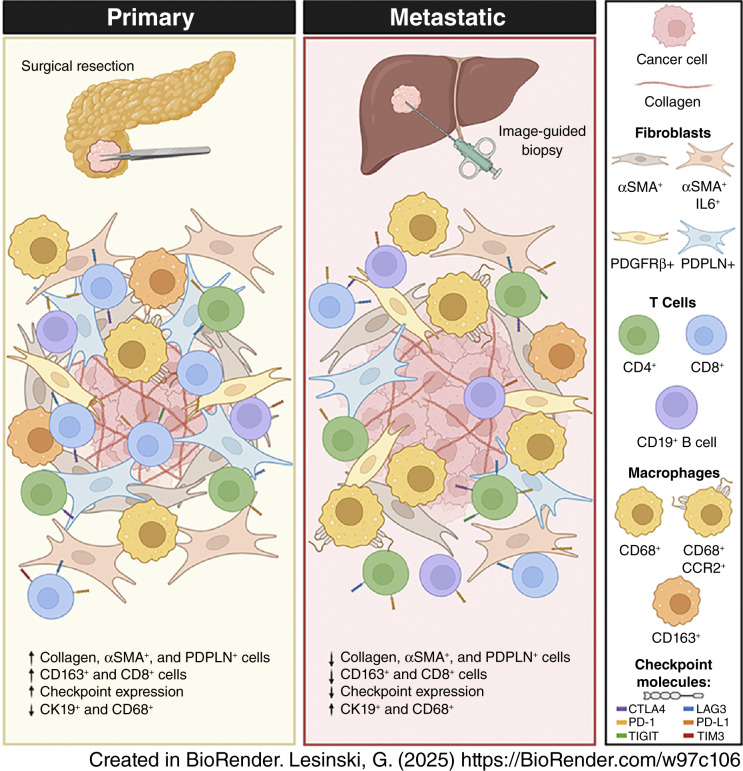

## Introduction

Patients with pancreatic ductal adenocarcinoma (PDAC) have 5-year overall survival of only 13% (1). When identified early, resection of primary tumors improves overall survival. However, about 80% of patients recur, usually presenting with metastases (2). The prevailing view is that most patients harbor micrometastatic disease in the liver or other organs, which is challenging to detect (3). These sobering scenarios underscore challenges that limit the efficacy of chemotherapy in patients with PDAC.

Immunotherapy does not have broad activity in patients with PDAC. This limited efficacy is attributed to multiple factors: (i) poor T-cell trafficking, (ii) limited neoantigens, (iii) a tumor microenvironment (TME) dominated by suppressive immune populations, and (iv) desmoplastic stroma that hinders antitumor immunity (4). Further complicating matters, most knowledge of human PDAC cellular composition and interactions comes from primary tumors rather than metastases (5–7). We posit that this limitation has biased selection of immune targets in clinical trials and may contribute to lack of efficacy in patients with metastatic disease. Although clinical efforts have focused on therapeutic combinations with PD-1/PD-L1 blockade, this approach has not yielded advances. Consequently, the role of other inhibitory immune checkpoints or costimulatory molecules remains an active area of interest. Emerging data using primary tumors from patients with PDAC suggest that alternative ligands such as lymphocyte-activation gene 3 (LAG3), T-cell immunoreceptor with Ig and ITIM domains (TIGIT), or T-cell immunoglobulin and mucin-domain containing-3 (TIM3) may be more abundant targets (8). Despite these efforts, there remains a need to tailor strategies in a data-driven manner, with focus given to immune features in metastatic rather than primary tumors.

Liver metastases are problematic given their propensity for spread and the inherently immunosuppressive nature of the liver. Preclinical studies demonstrate that liver metastases harbor abundant regulatory T cells (Treg; ref. 9) and attract activated Fas^+^CD8^+^ T cells from circulation, which undergo apoptosis following interaction with FasL^+^ macrophages (10). Hepatic stellate cells also promote Tregs and CD11b^+^Gr-1^+^ myeloid-derived suppressor cell (MDSC) differentiation (11). Additionally, studies have documented the propensity of hepatocytes to produce cytokines, such as IL6, to support a metastatic niche (12). Together, these observations, primarily from preclinical models, suggest the need for further insights into human PDAC liver metastases to rationally prioritize immunotherapy approaches for future clinical trials.

We hypothesized that PDAC tumors have distinct immunologic and stromal features depending on anatomic location. Using multiplex IHC (mIHC), we identified dominant immune cell populations in tumors from patients with primary and/or liver metastases. Metastatic tumors showed reduced T cells, fewer fibroblasts, and less collagen deposition compared with primary PDAC but were enriched in CD68^+^ macrophages (some CCR2^+^) and had a modest increase in CD19^+^ B cells. In contrast, CD163^+^ cells were more frequent in primary tumors. Spatial analyses revealed distinct cellular communities differing between sites. T-cell profiling showed fewer CD8^+^ T cells in metastases, whereas CD4^+^ and CD8^+^ T cells in primary tumors expressed higher RORγt and FoxP3. CyTOF validated these trends and enabled deeper phenotyping of B and T cells. Notably, both cell types coexpressed multiple inhibitory checkpoints, suggesting functional impairment. These protein-level data highlight key immune and stromal differences between primary and metastatic PDAC, providing insights to guide future immunotherapy strategies in the metastatic setting.

## Materials and Methods

### Patient demographics and tissue isolation

Fresh primary tumors were obtained from patients undergoing Whipple resection for histologically confirmed PDAC at the Winship Cancer Institute of Emory University following informed consent. Surgeries occurred between 2019 and 2024. Fresh liver punch biopsies of metastatic tissue were obtained from patients with histologically confirmed, inoperable disease enrolled in the clinical trials NCT03095781/WCI3321-16 and NCT04191421/WCI4463. As per clinical protocol, patients previously treated with αPD-1 and/or αPD-L1 were excluded. Enrollment occurred between 2017 and 2022. All patients had completed chemotherapy or other second-line regimens ≥2 weeks before biopsy and were off treatment at the time of sample acquisition. Demographics are reported in Supplementary Tables S1 and S2. Where possible, sex, race, ethnicity, and age were accounted for to accurately represent the larger population of patients, with both male and female patients included.

### Tissue preservation and quality

Samples were fixed in 10% neutral buffered formalin for 24 to 48 hours, processed, and embedded in paraffin. Formalin-fixed, paraffin-embedded blocks were sectioned at 4 μm for all hematoxylin and eosin (H&E), IHC, mIHC, and immunofluorescence (IF) stains. H&E-stained sections were pathologist-reviewed to confirm the presence of tumor and validate quality. Samples that did not meet pathologic requirements were excluded.

### IHC and mIHC

IHC and mIHC were performed on the DISCOVERY ULTRA autostainer (Roche Diagnostics). After deparaffinization, heat-induced epitope retrieval (HIER) was performed at 95°C using Cell Conditioning 1 (Roche, #06414575001) for 64 minutes. DISCOVERY Inhibitor (Roche, #7017944001) blocked endogenous peroxidase activity prior to iterative antigen staining. Each antibody was incubated for 40 minutes, followed by 12 minutes with OmniMap HRP secondary antibody (Roche, α-mouse #05269652001, RRID: AB_2885182 or α-rabbit #05269679001, RRID: AB_2811043), 16-minute incubation with an Opal fluorophore (Akoya Biosciences, Supplementary Table S3) diluted using 1X Amplification Diluent (Akoya Biosciences, #FP1609) or Disc. Diluent P.S.S. for Opal 780 only (Roche Diagnostics, #05266815001), and 8-minute denaturation at 93°C using Cell Conditioner 2 (Roche, #05279798001). Tissue was stained with individual (IHC) or a combination of (mIHC) antibodies directed against the markers listed in Supplementary Table S3. Antibody dilutions used Diamond Antibody Diluent (Cell Marque, #938B-09). Slides were counterstained with Spectral DAPI (Akoya Biosciences, #FP1490) for 16 minutes, cover-slipped (Mercedes Medical, #MER R2450), cleaned, and mounted using VECTASHIELD Antifade Mounting Medium (Vector Laboratories, #H-1000-10). Slides were cured for 24 hours in the dark at 4°C prior to imaging on the Vectra Polaris Automated Quantitative Pathology Imaging System. Ideal exposures were determined by averaging exposures across representative slides.

### Analysis of mIHC data

Whole-slide scans from the fluorescently labeled sections were analyzed using QuPath (v0.4.4.4, University of Edinburgh, RRID: SCR_018257; ref. 13). DAPI channel (C1) was used for cell segmentation (0.4967-μm pixel size, 6-μm background radius, background by reconstruction, 1-μm median filter radius, 0.5-μm sigma size, 3-μm^2^ minimum area, 400-μm^2^ maximum area, threshold 5, and 4-μm cell expansion area). Cells were classified using an adapted script (https://www.imagescientist.com/creating-a-classifier), with thresholds for each channel defined by visual intensity cutoffs to determine the number of positive cells for each fluorescent signal intensity or combination of signal intensities. All sections were analyzed simultaneously in batch mode. Data were exported and analyzed using Microsoft Excel (V. 16.93, RRID: SCR_016137) and GraphPad Prism (V. 10.4, RRID: SCR_002798). Percentages were calculated as the total cell detection for each florescent signal or combination of signals divided by the total number of cells. Densities were calculated as positive cells or combination of positive cells per tissue.

### Multiplex IF

Slides were baked overnight at 60°C, deparaffinized [2 × 10 minutes with xylene (08389-1, Polysciences), 2 × 5 minutes with 100% ethanol (UN1170, Koptec), 2 minutes with 96% ethanol, 2 minutes with 70% ethanol, and 5 minutes with dH_2_O], and subjected to HIER in 1 mmol/L EDTA (E5134-100G, MilliporeSigma) pH 9 for 30 minutes using a steamer. Slides were washed twice with PBS for 5 minutes and sequentially blocked with Human TruStain FcX (422302, BioLegend) for 60 minutes at room temperature at 1:200 in PBS and then using 5% donkey serum (GTX732205, GeneTex) in PBS for 60 minutes at room temperature. Primary antibodies listed in Supplementary Table S4 were applied in blocking buffer in excess and incubated twice overnight at 4°C. After PBS washes, secondary antibodies listed in Supplementary Table S4 were added at 1:500 in blocking buffer in excess for 60 minutes at room temperature. Slides were then blocked using Fab fragments [711-007-003, AffiniPure Fab Fragment Donkey Anti-Rabbit IgG (H + L), RRID: AB_2340587 and 715-007-003, AffiniPure Fab Fragment Donkey Anti-Mouse IgG (H + L), RRID: AB_2307338] at 1:100 in PBS, followed by additional Fcx blocking and then 5% donkey serum blocking, each for 60 minutes at room temperature. A second round of primary antibodies listed in Supplementary Table S4, in donkey serum buffer, was incubated twice overnight at 4°C, followed by secondary antibody application. Nuclei were counterstained with DAPI (D1306, Invitrogen) at a 1:1,000 in PBS for 60 minutes at room temperature or overnight at 4°C prior to mounting using VECTASHIELD mounting medium (H-1700-10, Vector Laboratories). Imaging was performed on a ZEISS Axioscan 7.

### Analysis of multiplex IF data

Single-cell fluorescence intensities were exported from QuPath (v0.6.0, University of Edinburgh, RRID: SCR_018257) and analyzed in R (v4.3.2, RRID: SCR_001905). Images with <500 cells were excluded. For each marker, per-image positivity gates were defined by two-class k-means clustering with a fallback of median + 3x median absolute deviation for unimodal distributions. Gates were adjusted using negative controls, and cells above final thresholds were designated positive. For the macrophage-specific panel (CD68, CCR2, and CD163), a background floor was set at the 99.5th percentile of unstained controls. For the cancer-associated fibroblast (CAF)–specific panel [cytokeratin 19 (CK19), podoplanin (PDPLN), platelet-derived growth factor receptor beta (PDGFRβ), and alpha smooth muscle actin (αSMA)], values were archsin-transformed (cofactor 150) to compress high signals with gates capped at the 99th percentile and floored by negative controls to maintain false positive rates (PDPLN 5%, PDGFRβ 10%, CK19 4%, and αSMA 1%). For both panels, false positives were validated on negative and single-stain controls. Per-image percentages and densities were summarized and compared between tissues via a Mann–Whitney test.

### Picrosirius Red staining and imaging

Picrosirius Red staining was performed using the Picrosirius Red Stain Kit for Connective Tissue (Abcam, ab150681) according to the manufacturer’s protocol. Briefly, reagents were equilibrated to room temperature and gently agitated. Sections were deparaffinized (as described above), hydrated in dH_2_O, incubated in Picrosirius Red solution for 60 minutes at room temperature, and washed twice in 0.5% acetic acid. Sections were dehydrated in absolute alcohol and mounted. Stained slides were imaged on a ZEISS Axioscan 7 (ZEISS) using both brightfield and polarized light.

### Analysis of Picrosirius Red staining

Whole-slide scans were analyzed using QuPath (v0.4.4.4, University of Edinburgh, RRID: SCR_018257; ref. 13). A pixel classifier was trained to threshold polarized signal and applied uniformly across samples. Data were exported and analyzed in Microsoft Excel (V. 16.93, RRID: SCR_016137) and GraphPad Prism (V. 10.4, RRID: SCR_002798). The percent of Picrosirius Red–positive area was calculated relative to total tissue area.

### Single-cell spatial analysis

Whole-tissue scans underwent quality control in QuPath prior to export of single-cell metadata. For fractured tissue, only the largest intact piece was analyzed; unsuitable regions were excluded. Cell neighborhoods, cell-to-cell interactions, cell colocalization, spatial heterogeneity, definition of tumor margins, and quantification of noncancer cell populations relative to tumor structures were analyzed with SPIAT from SpatialExperiment (14, 15) in R (v4.2, RRID: SCR_001905). Tissue mapping was performed in Python (v3.9.18, RRID: SCR_008394). Analyses were conducted per tissue to assess heterogeneity and pooled across primary or metastatic groups for comparison. Marker intensities from QuPath exports were treated as gene expression–like values, with cell coordinates and phenotypes as metadata. Cell communities were identified using the Spinglass algorithm in R with seed 123 for reproducibility (16). Networks were constructed with nodes (percent cell type) and edges (minimum intercellular distance), and modularity optimization grouped cells into communities. Communities were visualized as shaded bubbles behind nodes/edges, allowing systematic categorization of spatially associated cell types. Analysis of variables considered that most functions are independent of each other. Only tissues with clear tumor–stroma boundaries were included for infiltration analysis.

### Tissue digestion

Fresh tissue was dissociated using the gentleMACS system (gentleMACS, Miltenyi Biotec, #130-093-235) with the “tough” tumor protocol. The cut tissue (2–4 mm) was placed in C-tubes (Miltenyi Biotec, #130-093-237) containing RPMI-1640 and enzymes H, R, and A (Miltenyi Biotec, #130-095-929). Samples underwent two cycles of mechanical dissociation (program h_tumor_01) with the gentleMACS dissociator, followed by 30-minute incubation at 37°C with shaking, and one additional dissociation cycle. Single-cell suspensions were filtrated through 70-μm filters, centrifuged at 1,700 rpm for 5 minutes, and resuspended in PBS. Suspensions containing ≤2 × 10^6^ cells were processed for CyTOF staining.

### Single-cell mass cytometry staining

Viability was assessed by incubating cells with Cell-ID Cisplatin (Fluidigm Sciences, #201195) in PBS, quenching with Maxpar Cell Staining Buffer, and washing twice. Surface and intracellular staining was performed per the manufacturer’s protocol (Fluidigm Sciences) with antibodies listed in Supplementary Table S5. Cells were washed, fixed, permeabilized, and stained with intracellular markers. Following intracellular staining, cells were washed and incubated with Cell-ID Intercalator-Ir 125 μm (Fluidigm Sciences, #201192B). Samples were acquired on a Helios mass cytometer (Fluidigm Sciences), and raw flow cytometry standard (FCS) files were normalized using the CyTOF software normalizer algorithm. Single-cell mass cytometry data were analyzed as described below.

### Single-cell mass cytometry data analysis

Normalized FCS files were analyzed in FlowJo (v10.10, BD Biosciences, RRID: SCR_008520) and Cytobank (v10.6, RRID: SCR_014043; ref. 17). Cleanup followed Fluidigm technical notes (PN 400248 B1): normalization beads were excluded, Gaussian parameters (residual, center, offset, and width) removed merged ion clouds, and event length filtered abnormal pulses. Live cells were gated as cisplatin negative, and intact singlet nucleated cells were identified by DNA1/DNA2 iridium intercalator gates. Samples with ≥500 live DNA2^+^ cells were retained (primary, *n* = 12; metastatic, *n* = 23). Gating strategies are shown in Supplementary Figs. S1–S5. Cleaned FCS files were exported from FlowJo and uploaded into Cytobank for high-dimensional clustering analysis. DNA2^+^ cells were analyzed with optimized parameters for T-distributed stochastic neighbor embedding (opt-SNE; perplexity = 30; 1,000 iterations; 35 markers; and seed = 1,845,947,262; ref. 18) using equal event sampling. opt-SNE plots were generated for individual or concatenated samples (primary vs. metastatic) using Cytobank Illustration Editor. Manual gates were informed by healthy donor peripheral blood mononuclear cells.

### Statistical analysis

Statistical significance was analyzed with GraphPad Prism software (v10.4, RRID: SCR_002798). Significance was defined as *P* < 0.05. Comparisons between two groups used the unpaired two-tailed Mann–Whitney test (for nonnormal data) or Wilcoxon test (for matched samples). For multiple comparisons, two-way ANOVA with Šídák or Bonferroni *post hoc* analysis was applied. Full details are found in the figure legends.

### Ethics approval

Surgically resected tissue was obtained according to Emory University Institutional Review Board (IRB) protocol IRB00006401 [principal investigator (PI): Krasinskas, last approved: February 12, 2024] and protocol IRB00087397 (PI: Alese, last approved December 20, 2023). The NCT03095781/WCI3321-16 clinical trial was conducted according to Emory University IRB protocol IRB00087397 (PI: Alese, last approved December 20, 2023). The NCT04191421/WCI4463 clinical trial was conducted according to Emory University IRB protocol IRB000105616 (PI: Alese, last approved July 12, 2024). All studies were approved by the Emory University IRB and conducted in accordance with the Declaration of Helsinki and U.S. Common Rule. Written informed consent was obtained from all patients prior to tissue collection.

## Results

### Primary and metastatic PDAC harbor distinct cellular compositions

**Figure 1.**
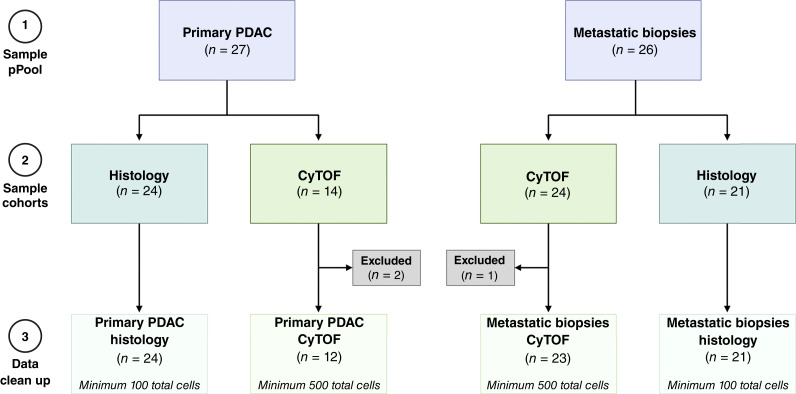
Patient sample workflow: acquisition, processing, and data analysis. Flow chart illustrating (1) the total number of samples acquired per tumor type, (2) the number processed via histology or CyTOF, and (3) the final number included for data analysis in this study based on minimum cell count criteria. Created in BioRender. Lesinski, G. (2025) https://BioRender.com/w97c106.

We posited that cellular features of primary and metastatic PDAC tumors in patients differ substantially. To test this, we interrogated primary resected PDAC tumors from consented patients (*n* = 27) and from image-guided core needle pretreatment biopsies of patients with liver metastases in a clinical trial (*n* = 26; Fig. 1). To minimize potential artifacts introduced by surgical resection, we ensured that all samples underwent quality control prior to analysis. Specifically, we evaluated viability and cellular composition to minimize sampling artifact. Patient demographics, treatment history, and anatomic site data are in Supplementary Tables S1 and S2. All patients received prior therapy, typically chemotherapy, and completed systemic therapy at least 2 weeks before resection or biopsy. Twenty-four primary PDAC tumors and 21 metastases were of sufficient quality for mIHC (Supplementary Tables S6 and S7). A portion of fresh tissue was digested into a single-cell suspension and underwent CyTOF analysis, yielding samples with sufficient numbers of cells for subsequent analysis in 12 primary and 23 metastatic tumors (Supplementary Tables S6 and S7). Two primary and one metastatic samples were excluded from CyTOF analysis because of insufficient cell viability.

**Figure 2.**
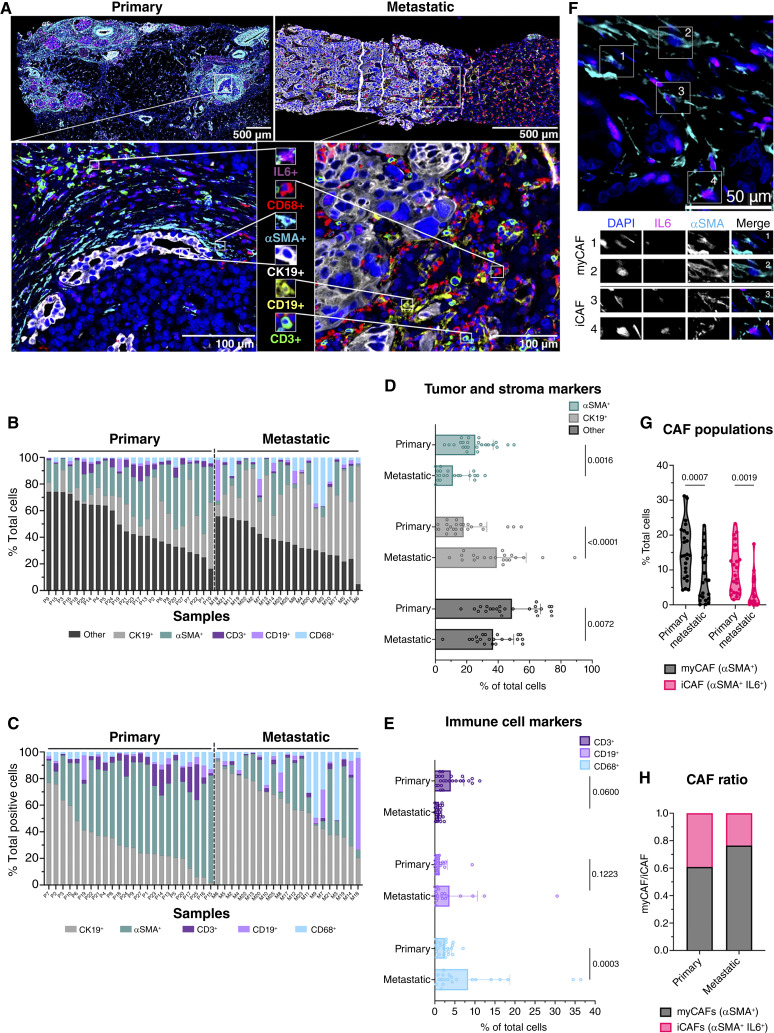
Primary and metastatic PDAC tumors have distinct cellular compositions. **A,** Representative fluorescent mIHC images of primary and metastatic tumors. Antibody panel includes IL6 (pink), CD68 (red), αSMA (cyan), CK19 (white), CD19 (yellow), and CD3 (green), with representative cells shown. Scale bar, top, 500 μm; bottom, 100 μm. **B** and **C,** Stacked bar graphs show the distribution of CK19^+^, αSMA^+^, CD3^+^, CD19^+^, CD68^+^, and DAPI^+^CK19^−^αSMA^−^CD3^−^CD19^−^CD68^−^ (other) cells in each patient sample as percent of total cells (**B**) and total phenotyped cells (**C**). Samples arranged from high to low based on other and CK19^+^ cells, respectively. **D **and **E, **Bar graphs quantify subsets separated into tumor and stroma markers (**D**) and immune cell markers (**E**), shown as a percentage of total cells (*P* values indicated). **F,** Representative image of myCAF and iCAF populations in stroma-rich tissue (top) with selected CAFs highlighted (myCAF labeled, 1–2; iCAF labeled, 3–4) in single channel DAPI, IL6, and αSMA images (bottom). Scale bar, 50 μm. **G,** Truncated violin plots quantify myCAF and iCAF populations as a percentage of total cells. **H,** Stacked bar graph shows the ratio of CAF populations in tumor tissue. Sample sizes: primary, *n* = 24; metastatic, *n* = 21. **D, E, **and **G,** Statistical analysis was performed using two-way ANOVA.

mIHC analysis revealed distinct cell compositions between primary and metastatic tumors (Fig. 2A; Supplementary Fig. S6; Supplementary Table S3). Notably, the immune contexture of primary and metastatic tumors was strikingly different (Fig. 2B–E). For instance, metastatic tumors were nearly devoid of T cells (Fig. 2B, C, and E). Although detectable, these cells comprised only a fraction of those within primary tumors. In contrast, CD68^+^ cells made up a large proportion of cells in metastatic tumors (primary, 2.44% ± 1.72% vs. 8.29% ± 10.4; Fig. 2E). Interestingly, CD19^+^ B cells were present in a subset of metastases but trended toward lower prevalence in primary tumors (Fig. 2E). As a salient feature of PDAC is a desmoplastic stroma populated by CAFs, we quantified αSMA^+^ cell frequency in the TME as a marker of activated CAFs. These cells were more frequent in primary versus metastatic tumors (25.5% ± 11.3% vs. 11.2% ± 10.6 total nucleated cells, respectively; Fig. 2B–D). Subclassification of CAFs using mIHC data, which identified myofibroblast-like CAFs (myCAF; αSMA^+^IL6^−^) and inflammatory CAFs (iCAF; αSMA^+^IL6^+^; Fig. 2F), revealed predominant myCAFs at both sites. However, both myCAF and iCAF were more infrequent in metastases (Fig. 2G). On a per-cell basis, however, the ratio of myCAF to iCAF was elevated in metastatic compared with primary lesions (Fig. 2H). These findings were confirmed by evaluating cell density of each population relative to tissue area (Supplementary Fig. S7A–S7H). Finally, high IL6 expression was observed across tumor sites (Supplementary Fig. S7I and S7J), suggesting a widespread protumor role within these tumors.

### Heterogeneity in fibroblasts and macrophages between primary and metastatic PDAC

**Figure 3.**
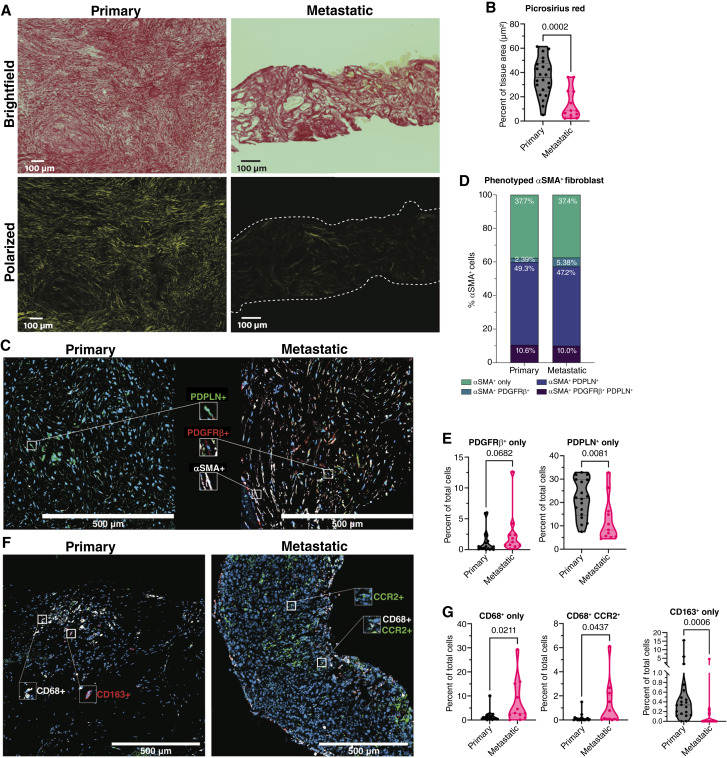
mIF confirms that fibroblast and macrophage populations differ between primary and metastatic PDAC. **A,** Representative images of Picrosirius Red–stained samples under brightfield and polarized light. Scale bar, 100 μm. **B,** Truncated violin plot quantifying percentage tissue area positive for Picrosirius Red staining. Sample size: primary, *n* = 23; metastatic, *n* = 13. **C,** Representative mIF fibroblast panel detecting αSMA (white), PDGFRβ (red), and PDPLN (green). Scale bar, 100 μm. **D,** Stacked bar graph showing the percent of total αSMA^+^ phenotyped cells in primary and metastatic tumors (not significant). **E,** Truncated violin plot quantifying PDGFRβ^+^-only and PDPLN^+^-only cells. Sample size: primary, *n* = 18; metastatic, *n* = 11. **F,** Representative mIF macrophage panel detecting CD68 (white), CD163 (red), and CCR2 (green). Scale bar, 500 μm. **G,** Truncated violin plots quantifying CD68^+^-only CD68^+^CCR2^+^ and CD163^+^-only as a percentage of total cells. Sample size: primary, *n* = 20; metastatic, *n* = 12. **B, E, **and **G,** Statistical analysis was performed using Mann–Whitney test; **D,** statistical analysis was performed using two-way ANOVA.

To better characterize stromal features, Picrosirius Red staining revealed significantly lower collagen fiber content in metastatic tumors compared with primary tumors (Fig. 3A and B; *n* = 23 and *n* = 13, respectively), consistent with trends observed in αSMA expression (Fig. 2). Multiplex IF (mIF) analysis of αSMA, PDGFRβ, and PDPLN (Fig. 3C; Supplementary Table S4; primary, *n* = 18; metastatic, *n* = 11) showed no significant differences in CAF subpopulations coexpressing PDGFRβ or PDPLN within the αSMA+ compartment across sites (Fig. 3D; Supplementary Fig. S8A–S8D). Most αSMA+ cells expressed either αSMA alone or coexpressed PDPLN, whereas PDGFRβ+ and dual PDGFRβ+/PDPLN+ populations were less frequent. Notably, PDGFRβ+ fibroblasts trended higher in metastatic tumors, whereas PDPLN+ fibroblasts were more abundant in primary tumors (Fig. 3E; Supplementary Fig. S8E and S8F). A subset of samples (*n* = 10 primary and *n* = 10 metastatic) was also stained for fibroblast activation protein (FAP), a potential marker of distinct CAF populations in PDAC (19). FAP^+^ cells were evident throughout the tumors, including ductal regions, and were equivalent in frequency between primary and metastatic tumors (Supplementary Fig. S8G–S8I).

In contrast to CAFs, CD68^+^ cells were abundant in metastases, prompting us to further define subsets within CD68^+^ macrophages. We evaluated CCR2 and CD163 via mIF (Fig. 3F; Supplementary Table S4; primary, *n* = 20; metastatic, *n* = 12). CD68^+^ macrophages were again higher in metastatic tumors, consistent with prior findings using mIHC methodology, and a proportion coexpressed CCR2 (Fig. 3G; Supplementary Fig. S8J and S8K). In contrast, primary tumors harbored higher proportions of CD163^+^ macrophages when compared with liver metastases, whereas CCR2 coexpression on these cells was negligible (Fig. 3G; Supplementary Fig. S8L and S8M).

### Differences in immune cell distribution and proximity to CK19^+^ cells between primary and metastatic PDAC

**Figure 4.**
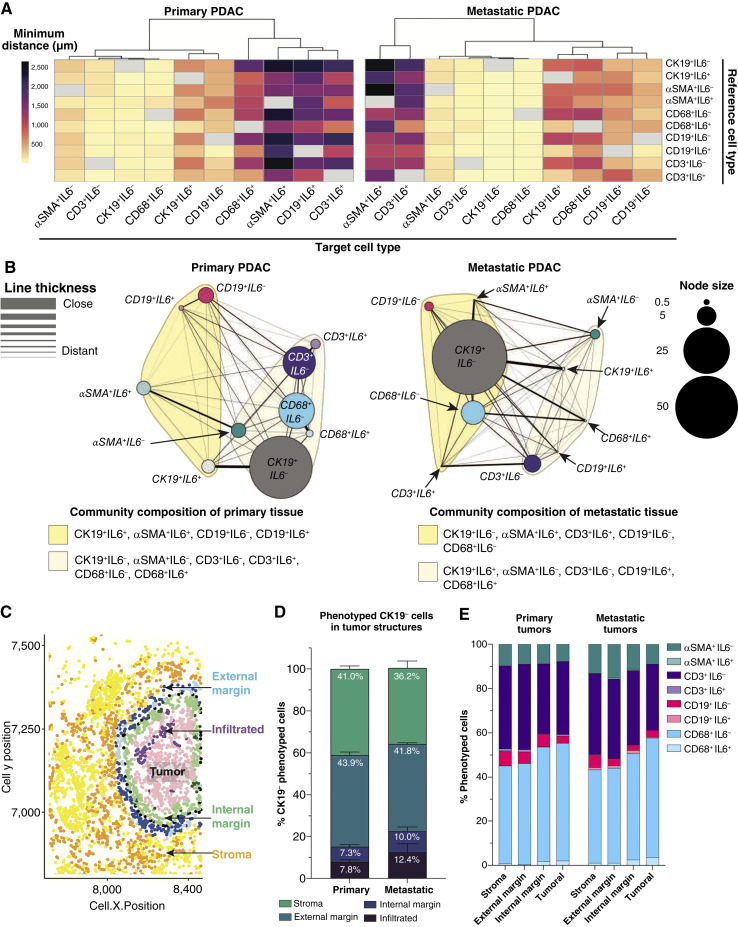
Cellular organization around CK19^+^ cells exhibit marked differences between primary and metastatic PDAC. **A,** Minimum distances measured (μm) between reference cell (*y*-axis) and target cell (*x*-axis) in primary and metastatic PDAC tissue represented as heatmaps with hierarchical clustering of target cell types (not significant). **B,** Network diagrams of primary and metastatic PDAC tissue with node size proportional to cell frequency and distances between cell types represented as line thickness. Cloud coloring around node groups represents communities. **C,** Representative dot plot depicting four tumor structure regions, namely stroma, external margin, and internal margin, infiltrated into the tumoral region. **D** and **E, **Stacked bar graphs indicating the percent of total CK19^−^ phenotyped cells (**D**) and identified cell subsets in each tumor structure of primary and metastatic PDAC tissue (not significant; **E**). Sample sizes: spatial analysis: primary, *n* = 24; metastatic, *n* = 20; tumor structure analysis: primary, *n* = 20; metastatic, *n* = 10. **A, **Statistical analysis was performed using two-sided Wilcoxon rank-sum test; **D** and **E,** statistical analysis was performed using two-way ANOVA.

To investigate distribution and organization of cells in primary and metastatic PDAC, *x*- and *y*-coordinates of phenotypically defined cell populations extracted from whole-tissue images (Fig. 2) were mapped after mIHC for all samples passing quality control. The median and minimum distances between all cell types were measured in both primary (*n* = 24) and metastatic (*n* = 20) tissues, showing little difference in overall dispersal (Supplementary Fig. S9A and S9B). To focus on cellular interactions, minimum distances (μm) between phenotyped cells were obtained (Fig. 4A; Supplementary Table S8) and summarized in a heatmap with hierarchical clustering. We found that αSMA^+^IL6^−^, CD19^+^IL6^+^, and CD68^+^IL6^−^ cells localized closer to CK19^+^IL6^−^ cells in metastatic PDAC than primary tumors (Supplementary Fig. S9C). Consistent with other studies and phenotypic properties of iCAFs (4), αSMA^+^IL6^+^ cells were distant from primary tumors but closer to CK19^+^IL6^−^ cells in metastases. In primary PDAC, IL6^+^ cells were positioned farther from CK19^+^IL6^−^ cells than IL6^−^ cells. In metastatic tissue, only CD3^+^IL6^+^ cells were more distant from CK19^+^IL6^−^ cells (Fig. 4A; Supplementary Table S8). Given the greater abundance of CD68^+^ and CD19^+^ cells in metastatic lesions, we focused on spatial relationships with each as reference cell types. These data indicated that CD19^+^IL6^−^ cells reside closer to CK19^+^IL6^−^ cells in metastatic tissue (Supplementary Fig. S9D). Across both sites, CD19^+^ cells were most closely associated with CD68^+^IL6^−^ and CD3^+^IL6^−^ cells but remained distant from αSMA^+^IL6^−^ and IL6 coexpressing cells. In metastatic PDAC, CD68^+^IL6^−^ cells localized closer to CK19^+^IL6^−^ and αSMA^+^IL6^−^ cells. At both sites, CD68^+^IL6^−^ cells closely localized with CK19^+^IL6^−^ and CD3^+^IL6^−^ cells (Supplementary Fig. S9E).

To summarize cell dispersion patterns, directional cell-to-cell distances and percentages were clustered as a network diagram, with closely associated cells identified as communities (Fig. 4B). Communities containing CK19^+^ cells varied between tumor types. In primary PDAC, CK19^+^IL6^−^ cells clustered with αSMA^+^IL6^−^ fibroblasts, CD3^+^ T cells, and CD68^+^ macrophages. In contrast, metastatic PDAC showed CK19^+^IL6^−^ cells grouping with αSMA^+^IL6^+^, CD3^+^IL6^+^, CD19^+^IL6^−^, and CD68^+^IL6^−^ cells, suggesting a shift in cancer–immune interactions during metastasis. Notably, in primary tumors, CD19^+^ B cells were linked to CK19^+^IL6^+^ and αSMA^+^IL6^+^ cells, whereas in metastases, a distinct cellular community emerged, composed of CK19^+^IL6^+^, αSMA^+^IL6^−^, CD3^+^IL6^−^, CD19^+^IL6^+^, and CD68^+^IL6^+^ cells.

Tissue architecture was assessed by the ratio of peritumoral cells (CK19^−^) to intratumoral cells (CK19^+^), excluding samples with ambiguous margins. This approach mapped distinct regions, such as intratumoral, internal, and external margins and stroma across 20 primary and 10 metastatic tissues (Fig. 4C). Quantification of CK19^−^ phenotype cells revealed similar fractions within each structure (Fig. 4D and E; Supplementary Fig. S9F; Supplementary Table S9). Subset analysis relative to mapped tumor regions (Supplementary Fig. S9E; Supplementary Table S10) showed CD3^+^ T cells predominated in stromal and external margins in both tumor types (Supplementary Fig. S9G–S9J), suggesting that T cells are less likely to penetrate the tumor. Notably, CD68^+^ cells were more abundant in internal margins and tumoral regions across sites (Supplementary Fig. S9G–S9J). Moreover, metastases contained more αSMA^+^ cells around external margins (Supplementary Fig. S9G–S9J). The overall presence of CD19^+^ B cells across tumor regions did not differ between disease sites (Supplementary Fig. S9G–S9J), suggesting that CD19^+^ B cells are prevalent throughout cancerous tissue.

### T cells from primary PDAC tumors express FoxP3 and RORγt

**Figure 5.**
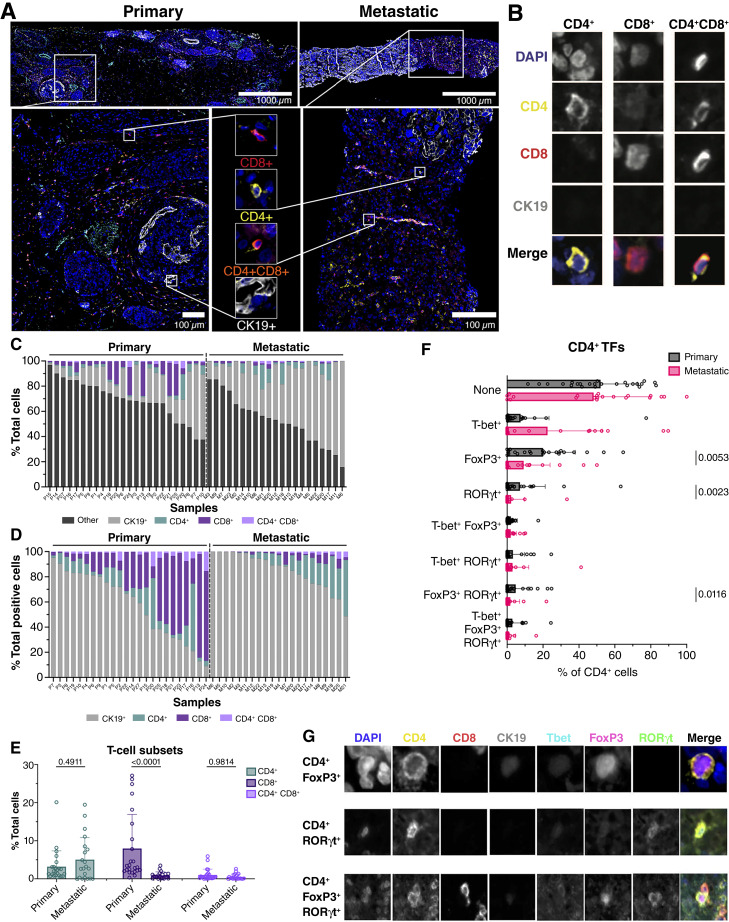
T cells in PDAC tumors are limited and express TFs with suppressive phenotypic properties. **A,** Representative images of mIHC staining in primary and metastatic tumors. Antibody panel detecting CD8 (red), CD4 (yellow), CK19 (white), and dual CD4^+^CD8^+^ cells (orange), with representative cells shown. Scale bar, top, 1,000 μm; bottom, 100 μm. **B,** Representative single-channel images of CD4^+^, CD8^+^, and CD4^+^CD8^+^ cell subsets (DAPI, CD4, CD8, and CK19). **C** and **D, **Stacked bar graphs show the distribution of CK19^+^, CD4^+^, CD8^+^, CD4^+^CD8^+^, and DAPI^+^CK19^−^CD4^−^CD8^−^ (other) cells detected by mIHC in each patient sample as percent of total cells (**C**) and percent of total phenotyped cells (**D**). Samples arranged from high to low based on other and CK19^+^ cells, respectively. **E,** Bar graphs show the mean ± SD of each T-cell subset (*P* values indicated). **F,** Bar graphs show the mean ± SD of TF (T-bet, FoxP3, and RORγt)-positive CD4^+^ T-cell populations, represented as a percentage of total CD4^+^ T cells (*P* values of significant results indicated). **G,** Representative images of CD4^+^ T-cell subsets by single-channel DAPI, CD4, CD8, CK19, T-bet, FoxP3, and RORγt. Sample size: primary, *n* = 24; metastatic, *n* = 21. **E, **Statistical analysis was performed using two-way ANOVA; **F, **statistical analysis was performed using Mann–Whitney tests.

T-cell subsets were more rigorously evaluated using mIHC (Fig. 5A and B; Supplementary Fig. S6; Supplementary Table S3). Consistent with data using the pan–T-cell marker CD3, very few CD8^+^ T cells were detected, with significantly greater frequency in primary compared with metastatic tumors (7.99% ± 8.95 and 0.90% ± 0.91 total nucleated cells, respectively, Fig. 5C and D). The limited CD8^+^ T cells in metastases precluded more detailed analysis of transcription factors (TF) or the small populations of T cells coexpressing CD4 and CD8 (Fig. 5C and D). CD4^+^ T cells were more frequent in primary (3.19% ± 4.16 total nucleated cells, Fig. 5D) and metastatic (5.04% ± 5.79 total nucleated cells, Fig. 5D) PDAC tumors, enabling characterization of TFs with greater confidence. We noted that FoxP3 and/or RORγt TFs were predominant in CD4^+^ T cells from primary tumors compared with metastatic tumors (Fig. 5E–G). A trend toward increased Th1-promoting T-bet expression in metastatic samples may suggest a more inflammatory phenotype in these cells. Although CD8^+^ T cells were fewer overall in tumors, T-bet expression was also predominant, regardless of tumor site. Although the overall frequency was quite limited, a fraction of these T-cell populations also expressed FoxP3 and RORγt (Supplementary Fig. S10A–S10C).

### CyTOF analysis reveals immunoregulatory features of lymphocyte populations in primary and liver-metastatic PDAC

**Figure 6.**
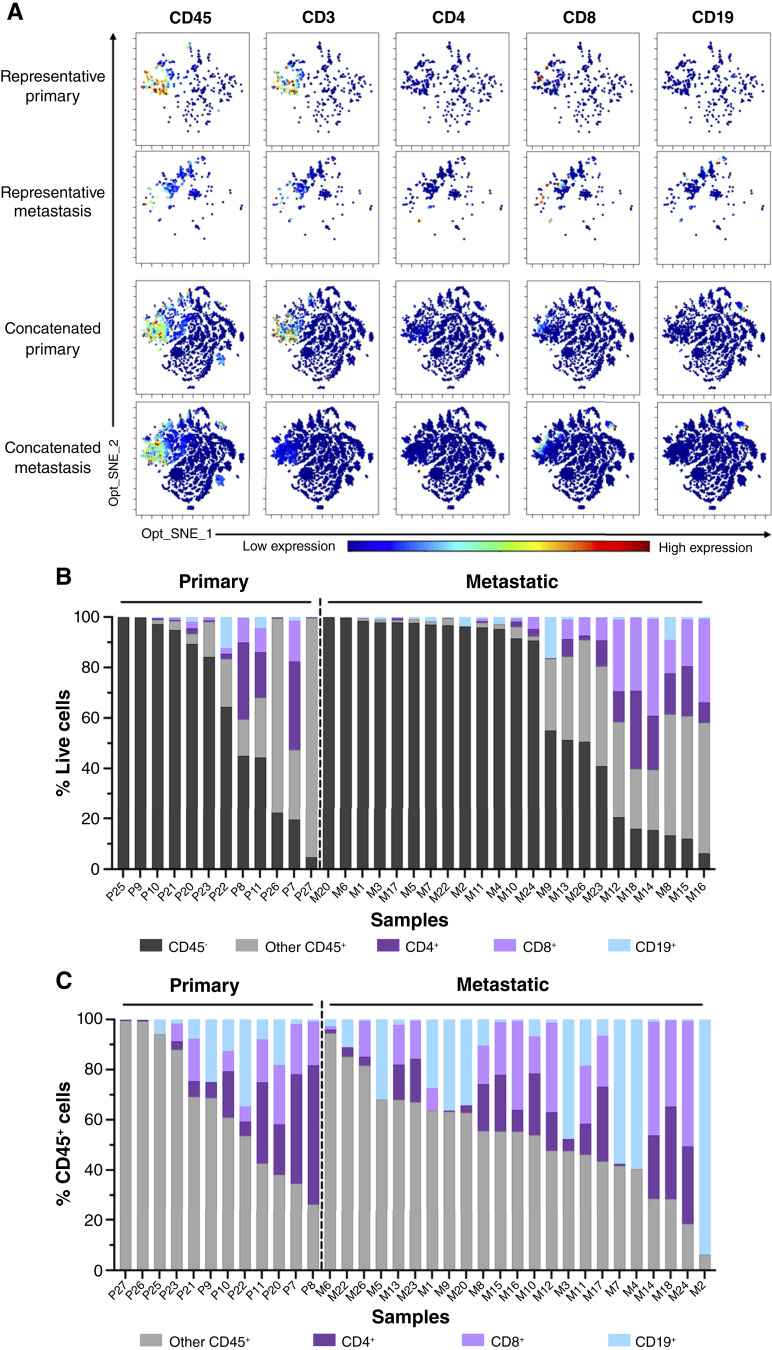
Single-cell analysis of lymphocyte populations by mass cytometry validates reduced T cells and increased B cells in liver metastases. **A,** Levels of CD45, CD3, CD4, CD8, and CD19 are displayed on individual, unsupervised Opt_SNE plots of live single cells in representative (top) and concatenated (bottom) primary and metastatic samples. **B,** Stacked bar graph showing the distribution of CD45^−^, other CD45^+^, CD4^+^, CD8^+^, and CD19^+^ cells as a percentage of total live cells per patient by mass cytometry. Samples arranged from high to low CD45^−^. **C,** Stacked bar graphs showing the distribution of other CD45^+^, CD4^+^, CD8^+^, and CD19^+^ as a percentage of total CD45^+^ cells per patient by mass cytometry. Samples arranged from high to low other CD45^+^. Sample size: primary, *n* = 12; metastatic, *n* = 23.

Mass cytometry is advantageous for providing single-cell resolution from small tissues. Given the overall paucity of T and B cells in PDAC, we used this technology with a comprehensive antibody panel to interrogate phenotype with greater scrutiny at the single-cell level (Supplementary Table S5). Following quality control, the analysis cohort consisted of fresh cell suspensions from 12 primary and 23 metastatic tumors. Clustering of concatenated data showed noticeable heterogeneity even among samples from the same tumor site (Fig. 6A). For example, primary samples P25 and P9 contained almost no immune cells, whereas CD45^+^ infiltration was high in samples P7 and P27 (Fig. 6B). Similarly, although most cells in metastases were CD45^−^, samples such as M8 and M9 harbored detectable CD45^+^ cells expressing CD4, CD8, and/or CD19 indicative of lymphocytes (Fig. 6C). Consistent with mIHC, primary tumors, on average, had more immune cells than metastases (36.23% ± 35.25% and 33.25% ± 36.79% CD45^+^ cells, respectively; Supplementary Fig. S11A; Supplementary Table S11). Lineage analysis showed CD8^+^ T cells and CD19^+^ B cells trended higher in metastatic versus primary tumors. Consistent with these data, CD8^+^ T cells and CD19^+^ B cells within metastases had more frequent positivity for the Ki67 proliferation marker (Supplementary Fig. S11B; Supplementary Table S11). Enzymatic tissue processing reduced cell yields across samples, limiting in-depth analysis of additional lineage markers and TFs; however, exploratory analyses were performed on samples meeting a ≥25 parent cell cutoff (Supplementary Figs. S1A–S1D, S2A–S2D, S3A–S3C, S12A–S12E, S13A–S13D; Supplementary Tables S11–S14).

CD19^+^ B cells were evident in PDAC tumors, particularly metastases. To characterize their phenotype, we analyzed tumor-infiltrating lymphocytes from patients using CyTOF. For robust analysis, a cutoff of 25 CD19^+^ events was applied. Six different phenotypically defined B-cell subsets were evaluated (Supplementary Fig. S14A; Supplementary Table S14), revealing several key findings: first, there was a trend toward a higher frequency of naïve B cells in primary tumors versus metastases. Although naïve B cells were present in primary tumors, metastatic tumors showed a trend toward a greater proportion of B cells with regulatory potential as indicated by PD-1 and/or PD-L1 expression. Also, a small population of CD27^+^ B cells, a marker of memory phenotype, was detectable in a subset of tumors although their frequency did not differ between primary and metastatic sites. B cells with extrafollicular or plasmablast phenotypes were rare across all samples. Finally, we assessed activation markers on B-cell subpopulations with regulatory potential, which showed high expression levels across both primary and metastatic tumors (Supplementary Figs. S4A–S4D, S14B, and S14C).

### T cells express inhibitory immune checkpoint receptors across disease sites

**Figure 7.**
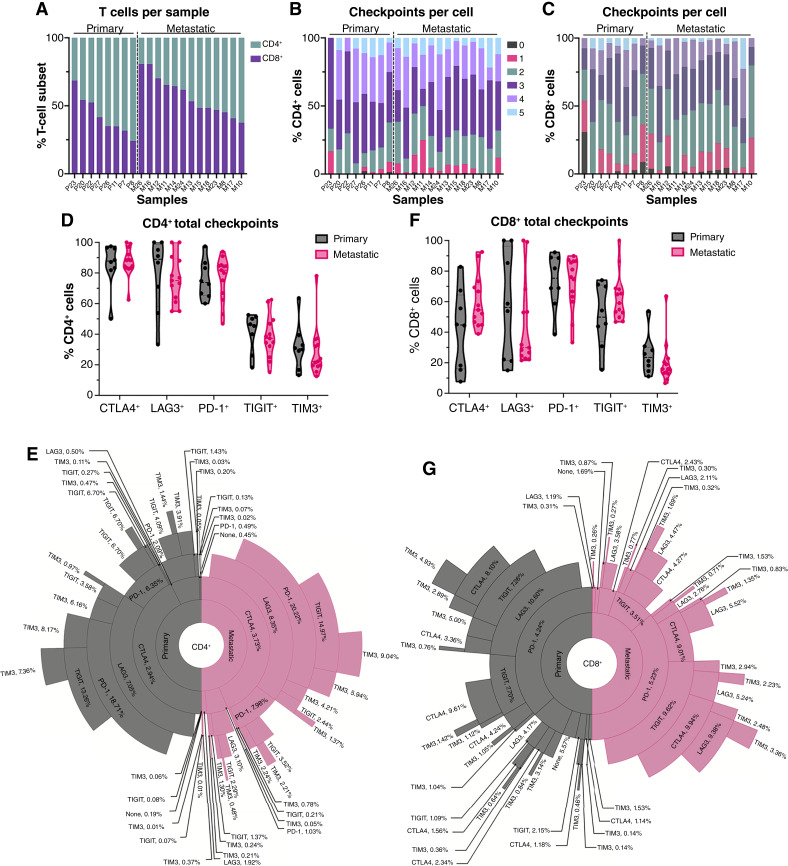
PDAC-associated T cells express prominent inhibitory immune checkpoint receptors regardless of disease site. **A,** Stacked bar graph of CD4^+^ and CD8^+^ T cells shown as the percentage of total T cells per sample. Samples are arranged from high to low CD8^+^ T cells. **B** and **C, **Stacked bar graphs show the distribution of checkpoint proteins detected on CD4^+^ T cells (**B**) and CD8^+^ T cells (**C**), indicating the number of checkpoints per cell. Samples arranged in the same order as **A**. **D,** Truncated violin plot quantifying total percentage of CD4^+^ T cells expressing CTLA4, LAG3, PD-1, TIGIT, and TIM3 checkpoint markers (not significant). **E,** Sunburst chart showing checkpoint marker combinations of CTLA4, LAG3, PD-1, TIGIT, and TIM3 found for CD4^+^ T cells, with percentages given for the terminal checkpoint in each combination. **F,** Truncated violin plots quantifying total percentage of CD8^+^ T cells (not significant). **G,** Sunburst chart showing checkpoint marker combinations of CTLA4, LAG3, PD-1, TIGIT, and TIM3 found on CD8^+^ T cells, with percentages given for the terminal check in each combination. Sample size: primary, *n* = 8; metastatic, *n* = 13. **D** and **F,** Statistical analysis was performed using Mann–Whitney tests.

We next evaluated the expression of targetable inhibitory checkpoint receptors on CD4^+^ and CD8^+^ T cells in primary and metastatic PDAC. These checkpoints included cytotoxic T lymphocyte–associated protein 4 (CTLA4), LAG3, PD-1, TIGIT, and TIM3 (Supplementary Figs. S5A–S5C and S15A). We applied a 25-cell cutoff to CD3^+^ T cells for robust data (Supplementary Table S15). This resulted in eight primary and 13 metastatic samples with sufficient events for comparison (Fig. 7A). Both CD4^+^ and CD8^+^ T cells from patients coexpress multiple inhibitory checkpoints (Fig. 7B and C). Further analysis indicated that CTLA4, LAG3, and PD-1 were most abundant on CD4^+^ T cells both in primaries and metastases (Fig. 7D). A trend toward higher TIGIT and TIM3 on CD4^+^ T cells was evident in primary versus metastatic tumors (41.26% vs. 38.38% of TIGIT, respectively, and 31.98% vs. 28.54%, respectively) although these were not statistically significant. Notably, checkpoints on CD8^+^ T cells were more variable. For example, CTLA4 and TIGIT trended toward higher expression in metastasis than primary samples (60.07% vs. 42.06% of CTLA4 and 62.84% vs. 50.61% of TIGIT, respectively). Boolean gating of these five markers was performed to assess the frequency of concurrent checkpoint expression combinations (Fig. 7E–G). In primary tumors, CTLA4, LAG3, and PD-1 coexpression, followed by CTLA4, LAG3, PD-1, and TIGIT coexpression, was dominant, encompassing >30% of CD4^+^ T cells. In metastases, these same checkpoint combinations dominated on >35% of CD4^+^ T cells (Fig. 7E; Supplementary Table S15). On CD8^+^ T cells, coexpression of LAG3 and PD-1, followed by CTLA4, PD-1, and TIGIT, was most prevalent in primary samples (encompassing >20%), whereas coexpression of CTLA4, PD-1, and TIGIT, followed by PD-1 and TIGIT, was prevalent in metastatic samples (encompassing >20%; Fig. 7G; Supplementary Table S16).

### mIHC analysis of matched primary and metastases validates trends of larger cohort analysis

Although remarkably rare and challenging to obtain in practice, we acquired matched primary and metastatic PDAC tissues from four patients with available archival specimens (Supplementary Fig. S16A). Although these tissues were included as part of the larger cohort, this separate analysis of matched patient tissues enabled a direct comparison of trends in TME changes by PDAC location. Consistent with findings from our unmatched patient cohort (Fig. 2), patient-matched metastases exhibited trends toward higher proportions of CK19^+^ and CD19^+^, with CD68^+^ cells consistently higher in all four patients analyzed (Supplementary Fig. S16B and S16C). In contrast, αSMA^+^ and CD3^+^ cells were more abundant in primary tumors (Supplementary Fig. S16B and S16C). Additionally, metastases had more frequent myCAFs and fewer iCAFs in three of four patients (Supplementary Fig. S16D). In these samples, CD8^+^ T cells trended to lower in metastatic PDAC, whereas there was no trend for CD4^+^ T cells (Supplementary Fig. S16E and S16F).

## Discussion

This study uncovers crucial differences in cellular composition of the TME between primary and metastatic PDAC as summarized in the graphical abstract. Although a handful of elegant studies have compared either normal and cancerous tissue (8) or profiled primary and metastatic PDAC using single-cell transcriptomics (20–27), our study provides novel, high-dimensional protein-level data across disease sites. Given the rarity and difficulty of obtaining metastatic PDAC biopsies for research, the observations from these samples are particularly valuable and informative.

It is important to note that many prior transcriptomic studies analyzed smaller patient cohorts and lacked protein-level validation. By contrast, our study leverages multiplexed imaging and mass cytometry to characterize lineage-specific markers across a broader set of patient tissues, offering a critical and complementary perspective to the existing literature. Although our protein-based panels include fewer phenotypic markers than transcript-based methods, they were purposefully designed to capture key immune and stromal populations in the PDAC TME and allowed rigorous cross-platform validation. Notably, the absence of certain immune subsets in our dataset, such as low T-cell frequencies, is consistent with prior reports focused on primary PDAC (8).

Among our findings, we observed a scarcity of T cells, especially in metastatic tumors. In contrast, CD68^+^ cells were more abundant in metastases and often coexpressed CCR2, suggesting origins from circulating monocytes recruited to tumors. Conversely, CD163^+^ cells were higher in primary tumors although overall frequencies were low. Other studies have documented elevated suppressive macrophages (26) or their corresponding gene signatures (25), aligning with our data. These macrophage patterns were accompanied by a trend toward increased CD19^+^ B cells, indicating that these populations may play a unique role in this setting. Conversely, CD8^+^ T cells were less frequent in metastases. Spatial analysis indicated that CD19^+^, CD68^+^, and CD3^+^ cells are present as “communities” in both primary and metastatic tumors, often localizing to tumor margins. In metastatic tumors, CK19^+^ cells associated closely with αSMA^+^, CD19^+^ IL6^+^, and CD68^+^ cells. Further analysis revealed that CD4^+^ T cells expressed RORγt and FoxP3 TFs, along with inhibitory checkpoint receptors, indicative of a suppressive phenotype. These data provide novel insights into unique cellular features in PDAC by disease site and provide rationale for prioritizing targeted and immunotherapy approaches aimed at restoring T cells in the metastatic PDAC TME, such as inhibitors of oncogenic Ras signaling or even adoptive cell transfer therapy.

The concept of whether CAFs promote or restrain PDAC progression has been a topic of controversy and interest. Many assumptions about interactions between CAFs and PDAC are from murine models or primary human tumors, with more limited data on their frequency or role in metastatic disease. Not surprisingly, primary PDAC harbored abundant αSMA^+^ and PDPLN^+^ cells, and consistent with other studies (28), these cells were also present in metastases, albeit less frequently in our cohort of heavily pretreated patients. Other CAF markers, such as PDGFRβ, trended in the opposite direction, underscoring CAF heterogeneity across different sites. However, we also considered FAP expression given reports that PDAC may harbor distinct FAP^+^ and αSMA^+^ subsets (19). Our analysis showed that FAP^+^ cells were ubiquitous, often present in regions of ductal adenocarcinoma, consistent with prior observations in PDAC tissues and cell lines (Supplementary Fig. S8G–S8I; ref. 29). Collagen deposition was also more pronounced in primary tumors, in line with αSMA and PDPLN findings. Together, these data suggest that strategies to deplete or modify stroma may be less impactful for patients with refractory metastatic disease and may help explain why clinical trials targeting this feature of PDAC have, to date, produced underwhelming results. Although these data are relevant, we acknowledge the inherent heterogeneity and plasticity of CAFs and the difficulty of distinguishing them from hepatic stellate cells (22).

Our observation that PDAC metastases harbored a greater proportion of CD68^+^ cells while primary tumors had trends toward more CD163^+^ cells was interesting. Data from both human tumors and murine models suggest that macrophages in PDAC represent sought-after targets to reverse suppressive immune features (3, 7, 25). Our data remain consistent with several other studies reporting the role of macrophages in PDAC (30–32) and suggest that PDAC reliance on macrophages may intensify as tumors progress. We speculate that this “macrophage amplification” may be fueled by inflammatory cytokines in liver or skew chemokine gradients to attract them efficiently. Indeed, our prior data with patient-derived CAFs revealed a prominent role for IL6, SDF1, GM-CSF, and others that shape macrophage biology (33). These data suggest that cytokine and chemokine circuits that foster macrophages, at the expense of dendritic cells, may further amplify disease progression and represent viable targets (34–37). Furthermore, a study by Lee and colleagues (12) demonstrated that IL6/STAT3 signaling leads to the production of serum amyloid A1 and A2 (SAA). This increase in SAA from hepatocytes favored a prometastatic niche in the liver. The fact that CD68^+^ cells predominated metastatic lesions, and T cells were few, supports other conclusions from work by Yu and colleagues (10). Using preclinical models, these studies showed that macrophages in liver metastases elicit Fas–FasL–mediated apoptosis of CD8^+^ T cells, eliminating them from the TME. Data herein are significant as, to date, macrophage-targeted therapeutic approaches, including CSF1R- and CCR2-/CCR4-directed therapies, have not made a dramatic impact in metastatic PDAC when combined with other approaches in clinical trials (38–40). Our data support these findings as there were numerically few CCR2^+^ macrophages quantified in tumors from our cohort of patients. Likewise, initial promising clinical data combining anti–PD-1 and chemotherapy with CD40 agonists unfortunately did not show superiority to anti–PD-1 and chemotherapy in a randomized phase II trial (41, 42). Given these disappointing outcomes with macrophage-targeted agents, deeper scrutiny of macrophage subsets in metastatic disease is warranted. For instance, future studies should comprehensively define dominant chemokine or cytokine receptors on these cells, and assess how these change with disease progression, to uncover optimal targeting.

The trend toward higher frequencies of CD19^+^ B cells in metastatic PDAC tumors was unexpected. Although other studies indicate that B cells are detectable, limited data on their nature in metastases were available (20, 22, 23, 43). More recently, a pioneering study from Pei and colleagues (27) used single-cell spatial transcriptomic approaches on matched primary and metastatic PDAC tissues from a rare cohort of 13 rapid autopsy patients, documenting that plasma cells are likely excluded from metastatic PDAC. They further elucidated that this exclusion occurred via a cooperation between basal-like PDAC cells and CAFs via the CXCL12/CXC4 axis. Although our mass cytometry panel was intentionally designed on T-cell markers, it permitted interrogation of some key proteins on B cells in select patients at single-cell resolution. Regardless of site, B cells displayed features consistent with immune suppression rather than stimulation. These data argue that B cells in tumors are unlikely to facilitate antigen presentation or productive antitumor responses. The idea that B cells support PDAC has inspired studies targeting Bruton tyrosine kinase to interfere with suppressive features (44). Other reports point to the presence of B cells with a regulatory phenotype in primary human PDAC, such as those producing IL10 or IL35 (45). It will therefore be of interest to delineate whether these B-cell features are also evident in metastases as a future objective.

In this study, we also had access to a unique set of matched samples from four patients, allowing us to evaluate the direct progression of the PDAC TME and validate trends from the larger cohort. Patients do not typically undergo biopsies of metastasis for routine care; thus, even a small cohort such as ours is quite rare and informative, often taking several years to accumulate from pathology archives. Although most cells evaluated trended similarly across all patients, the few that diverged may indicate differences in disease process within each patient or could simply be due to variability because of small sample size. Regardless of their limitations in number, these samples were valuable and informed our data.

Our data show sparse T-cell infiltration in primary PDAC, particularly CD8^+^ T cells, consistent with previous reports (46). Metastatic tumors had even fewer T cells, predominantly CD4^+^ lymphocytes suggesting that adoptive T-cell therapies may be worthwhile in this setting. TF profiling revealed increased FoxP3 and RORγt expression in CD4^+^ T cells in primary tumors, consistent with Th2/Th17 skewing (47, 48), and the presence of Tregs and Th17 cells is known to drive PDAC progression (49–51). In contrast, CD4^+^ T cells in metastases expressed more Tbet, indicating a more inflammatory TME. Notably, many CD4^+^ T cells lacked the expression of Tbet, RORγt, or FOXP3, suggesting a population of exhausted or dysfunctional T cells with downregulated TFs (52).

We also identified a small population of CD4^+^ CD8^+^ double-positive T cells, previously observed in other cancers but (53, 54), to our knowledge, reported here for the first time in PDAC tumors. The coexpression of suppressive TFs and inhibitory checkpoints on these and other T cells may contribute to immunotherapy resistance.

Consistent with prior findings in primary PDAC (8), we observed dominant LAG3 and PD-1 on both CD4^+^ and CD8^+^ T cells. These results support testing rational immunotherapy combinations, such as LAG3 blockade with myeloid-targeting approaches. Future studies using mIHC could validate these findings and expand spatial data on T-cell populations. Additional strategies, including cancer vaccines, may help mobilize tumor-specific T cells (55). Ultimately, improving T-cell trafficking, infiltration, and persistence remains critical for advancing PDAC immunotherapy.

This study provides a comprehensive protein-level analysis of the TME in primary and metastatic human PDAC. Although the small number of matched patient samples limits conclusions about individual disease progression, the findings offer valuable insights. Reliance on image-guided biopsies, which under-sample tumor heterogeneity compared with full resections, also constrains interpretation. As with other biopsy-based studies—including single-cell RNA sequencing—these results should be cautiously generalized (8, 22, 30, 56). Nonetheless, our analysis reveals distinct cellular relationships that can inform future research. In particular, the roles of macrophage and B-cell populations in metastases merit further investigation in larger cohorts.

We acknowledge limitations of mIHC/mIF in accommodating broad antibody panels and that our CyTOF panel focused primarily on T-cell markers, limiting insights across immune populations. Future studies should expand marker sets to better characterize the TME. Functional assays, such as cytokine profiling, T-cell receptor clonality, and *ex vivo* T-cell activation could clarify whether exhausted T-cell phenotypes correspond with impaired function. Furthermore, CK19 was used as a marker of malignant epithelial cells because of its established role in PDAC diagnostics. Despite these constraints, our findings reveal immune exhaustion patterns and redundant mechanisms of immunotherapy resistance, supporting prioritization of emerging targets on tumor-associated macrophages or MDSCs in combination immunotherapy.

Importantly, these data reflect the TME of pretreated and not treatment-naïve patients. Although this may limit mechanistic interpretation, heavily pretreated patients are most likely to enroll in trials of novel therapies. The absence of an independent validation cohort also limits generalizability and warrants cautious interpretation until replicated. Patients in this study also did not receive PD-1–/PD-L1–targeted immunotherapy, consistent with their limited efficacy in PDAC. Therefore, future studies including such samples could offer insights into therapy-induced TME changes. Finally, this study focused on liver metastases, a common site in PDAC with a distinct microenvironment. Future work should extend to other metastatic sites or tumor-draining lymph nodes to explore site-specific immune and stromal features.

## Supplementary Material

Supplemental Figure 1 Representative gating strategy for mass cytometry data

Supplemental Figure 2 Mass cytometry gating strategy of myeloid and NK cell populations

Supplemental Figure 3 Mass cytometry gating strategy of CD4+ and CD8+ T cell subpopulations

Supplemental Figure 4 Mass cytometry gating strategy of CD19+ cell subpopulations

Supplemental Figure 5 Mass cytometry gating strategy of inhibitory immune checkpoint receptors present on CD4+ and CD8+ T cell populations

Supplemental Figure 6 Single stain IHC validation of multiplex IHC panels

Supplemental Figure 7 Density of phenotyped cells from mIHC analysis validates total cell quantification

Supplemental Figure 8 Density of phenotyped cells from mIF analysis confirming total cell quantification

Supplemental Figure 9 Spatial analysis reveals distinct cellular distribution and proximity of immune cells to CK19+ cells in primary and metastatic PDAC tumors

Supplemental Figure 10 Transcription factor levels in CD8+ T cells reveals trends toward suppressive phenotypes

Supplemental Figure 11 Mass Cytometry quantification of lymphocyte populations and proliferation in primary and metastatic tissue

Supplemental Figure 12 Mass cytometry analysis of myeloid and NK cells in primary and metastatic PDAC

Supplemental Figure 13 Mass cytometry analysis of T cell subsets in primary and metastatic PDAC

Supplemental Figure 14 Mass cytometry analysis of B cell subsets and their activation markers in PDAC

Supplemental Figure 15 Opt_SNE plots of checkpoint marker expression on CD4+ and CD8+ T cells

Supplemental Figure 16 Matched primary and metastatic PDAC samples validates trends observed in the larger cohort analysis

Supplemental Table 1 Demographics of patients with primary tumors

Supplemental Table 2 Demographics of metastatic patients

Supplemental Table 3 Multiplex IHC antibodies

Supplemental Table 4 Additional multiplex IHC antibodies

Supplemental Table 5 CyTOF antibody panel

Supplemental Table 6 Primary sample cell count

Supplemental Table 7 Metastasis sample cell count

Supplemental Table 8 Distance between cells

Supplemental Table 9 Percent of cells in each tumor structure

Supplemental Table 10 Percent phenotyped cell in tumor structure

Supplemental Table 11 Immune cell populations

Supplemental Table 12 T cell counts

Supplemental Table 13 CD11b+ cell counts

Supplemental Table 14 CD19+ cell counts

Supplemental Table 15 CD4+ checkpoints

Supplemental Table 16 CD8+ checkpoints

## Data Availability

The data generated in this study are available from the corresponding author upon request. The datasets include patient information and immunoprofiling data obtained through mass cytometry (CyTOF), mIHC, mIF, and histologic analysis. Analytic code was adapted from previously published sources (14, 15), with minor data-specific modifications available from the authors upon request.
