# Supplementary material for: High-Dimensional Protein Analysis Uncovers Distinct Immunologic and Stromal Features in Primary and Metastatic Pancreatic Ductal Adenocarcinoma
Source: Cancer Res. 2025 Dec 19;86(7):1753–68. doi: 10.1158/0008-5472.CAN-25-1697 (PMC13044534; doi:10.1158/0008-5472.CAN-25-1697)
Supplement: Supplemental Figure 1 — Representative gating strategy for mass cytometry data [file can-25-1697_supplemental_figure_1_suppsf1.pdf]

Supplemental Figure 1

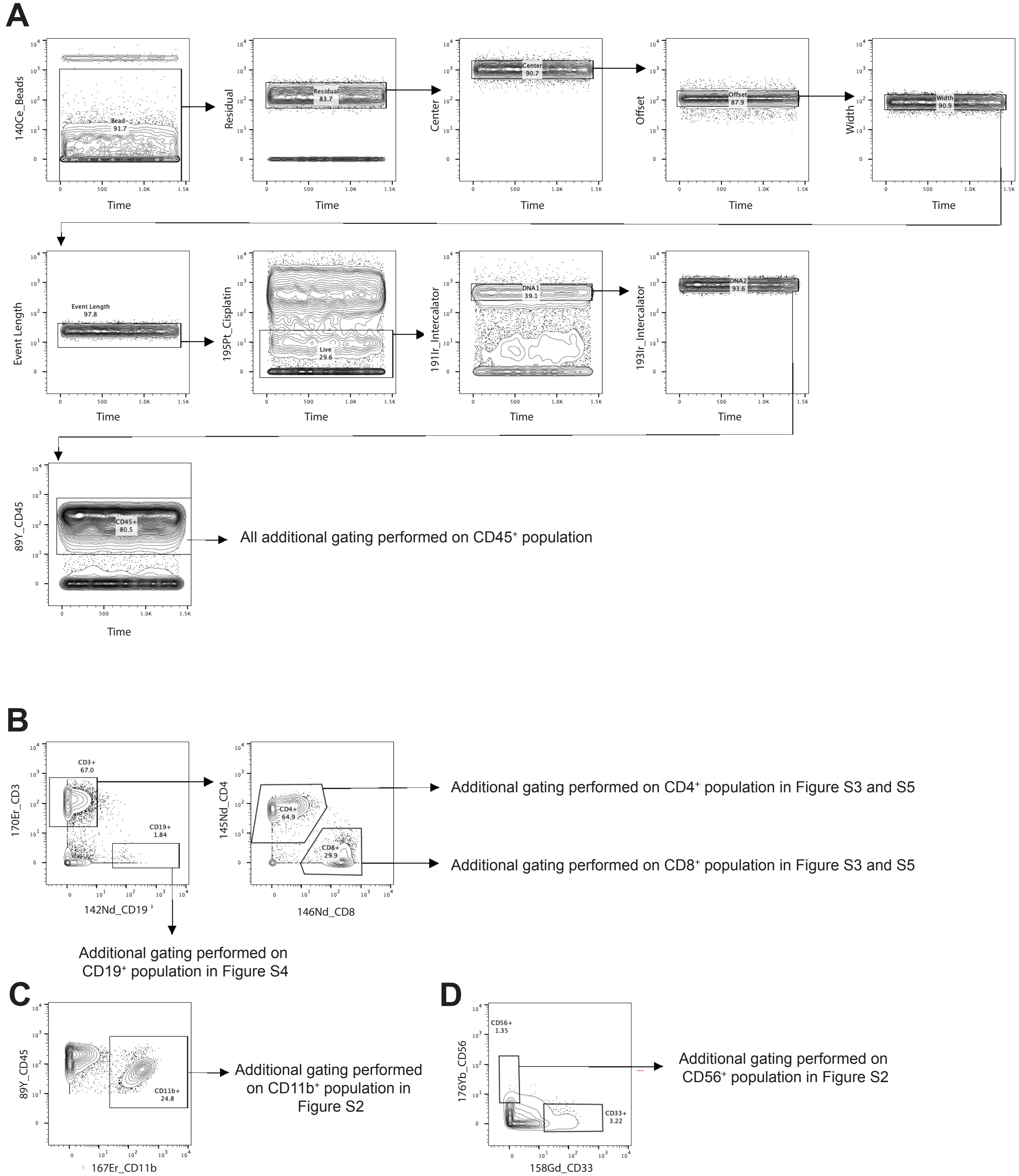

Supplemental Figure 1 Representative gating strategy for mass cytometry data. (A) $CD45^+$ cells gated from live single cell populations. (B) $CD3^+$ and $CD19^+$ cells identified from $CD45^+$ cells, with $CD4^+$ and $CD8^+$ subsets gated from the $CD3^+$ parent population. (C) Identification of $CD11b^+$ cells from $CD45^+$ cells. (D) Identification of $CD56^+$ cells from $CD45^+$ cells. Representative sample: P7.
