# Supplementary material for: High-Dimensional Protein Analysis Uncovers Distinct Immunologic and Stromal Features in Primary and Metastatic Pancreatic Ductal Adenocarcinoma
Source: Cancer Res. 2025 Dec 19;86(7):1753–68. doi: 10.1158/0008-5472.CAN-25-1697 (PMC13044534; doi:10.1158/0008-5472.CAN-25-1697)
Supplement: Supplemental Figure 2 — Mass cytometry gating strategy of myeloid and NK cell populations [file can-25-1697_supplemental_figure_2_suppsf2.pdf]

Supplemental Figure 2

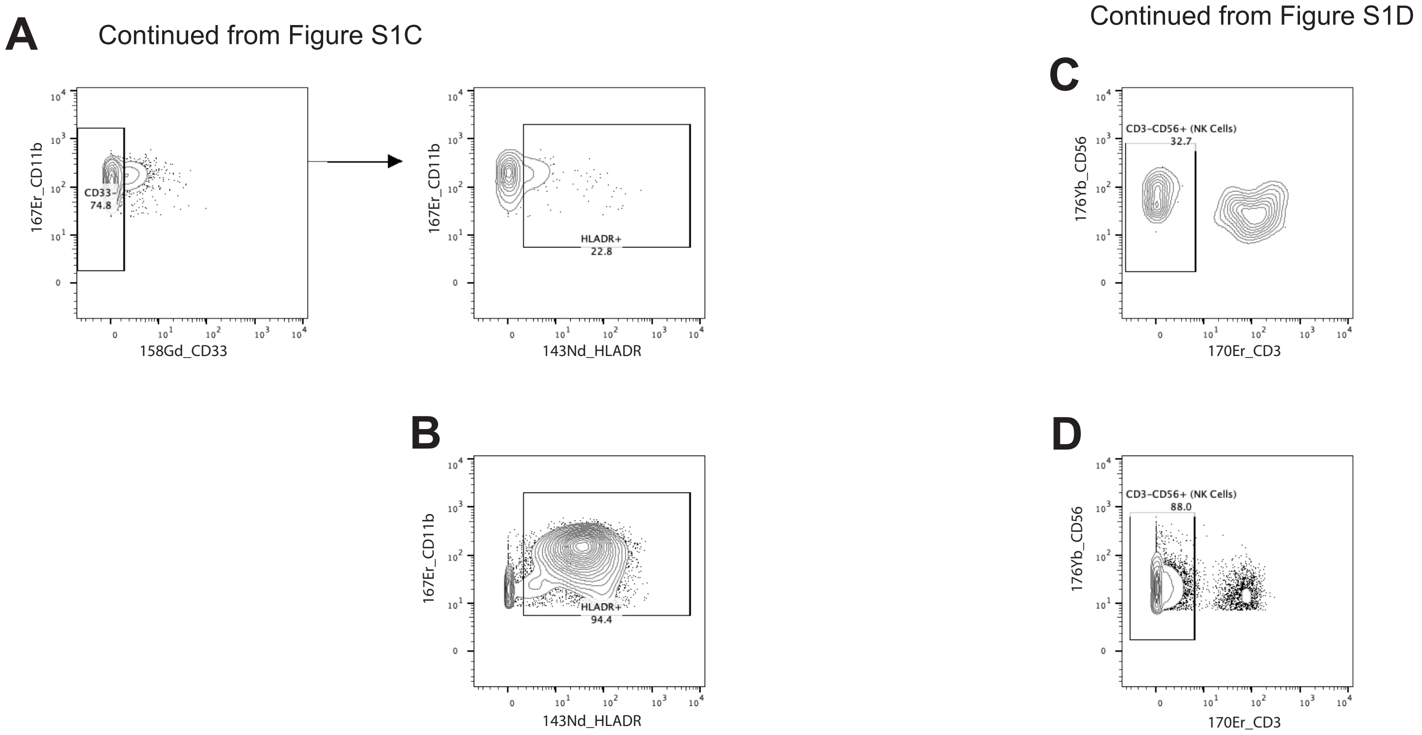

Supplemental Figure 2 Mass cytometry gating strategy of myeloid and NK cell populations. (A) Representative plots showing identification of $CD11b^+$, $CD33^+$, HLA-DR^- cells. Representative sample: M12. (B) Corresponding gating in healthy donor blood for comparison. (C) Representative plots showing identification of $CD3^-$ $CD56^+$ NK cells. Representative sample: M23. (D) Corresponding gating in healthy donor blood for comparison.
