# Supplementary material for: High-Dimensional Protein Analysis Uncovers Distinct Immunologic and Stromal Features in Primary and Metastatic Pancreatic Ductal Adenocarcinoma
Source: Cancer Res. 2025 Dec 19;86(7):1753–68. doi: 10.1158/0008-5472.CAN-25-1697 (PMC13044534; doi:10.1158/0008-5472.CAN-25-1697)
Supplement: Supplemental Figure 3 — Mass cytometry gating strategy of CD4+ and CD8+ T cell subpopulations [file can-25-1697_supplemental_figure_3_suppsf3.pdf]

Supplemental Figure 3

Continued from Figure S1B for CD4 cells

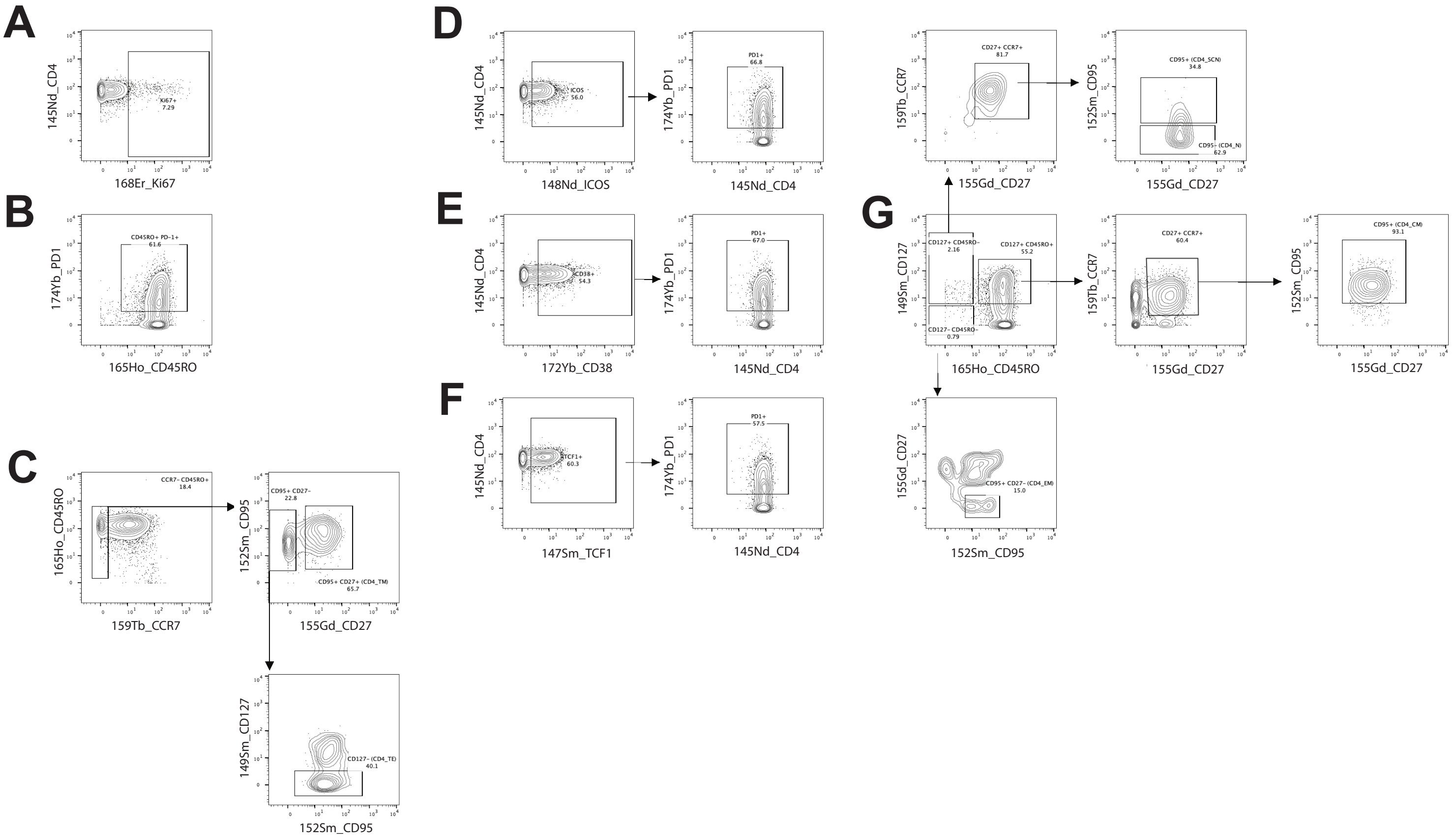

Continued from Figure S1B for CD8 cells

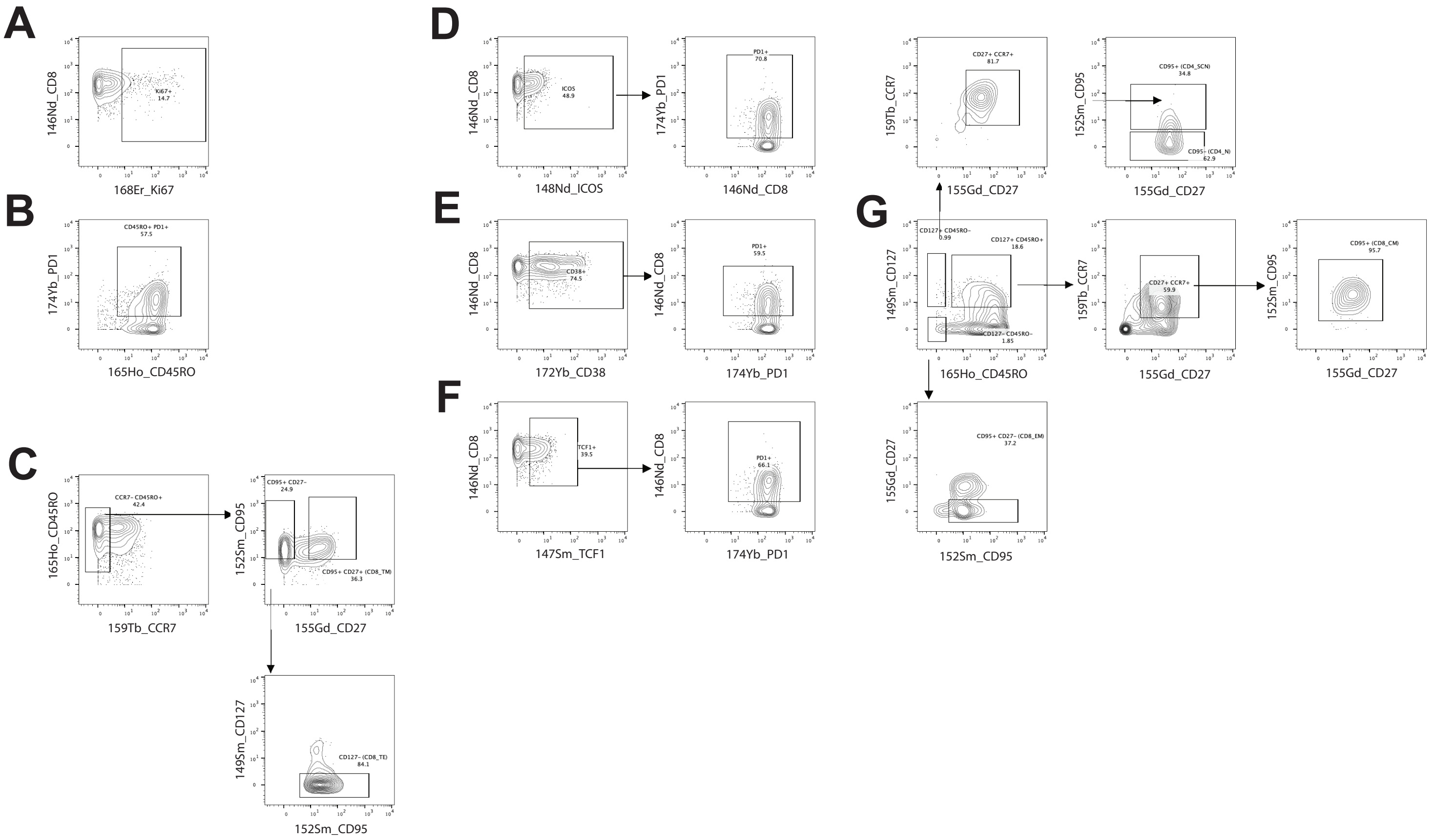

Supplemental Figure 3 Mass cytometry gating strategy of $CD4^+$ and $CD8^+$ T cell subpopulations. Subpopulations include (A) Ki-67^+, (B) $CD45RO^+PD\text{-}1^+$, (C) $CCR7^-CD45RO^+CD95^+CD27^-CD127^-$, terminal effector (TE) cells and $CCR7^-CD45RO^+CD95^+CD27^+$ transitional memory (TM) cells, (D) $ICOS^+PD\text{-}1^+$, (E) $CD38^+PD\text{-}1^+$, (F) TCF-$1^+PD\text{-}1^+$, and (G) $CD127^+CD45RO^-CD27^+CCR7^+CD95^+$ stem-like central memory (SCM) cells; $CD127^+CD45RO^-CD27^+CCR7^+CD95^-$, naïve (N) cells; $CD127^+CD45RO^+CD27^+CCR7^+CD95^+$, central memory (CM) cells; $CD127^-CD45RO^-CD27^-CD95^+$ effector memory (EM) cells. Representative sample: P7.
