# Supplementary material for: High-Dimensional Protein Analysis Uncovers Distinct Immunologic and Stromal Features in Primary and Metastatic Pancreatic Ductal Adenocarcinoma
Source: Cancer Res. 2025 Dec 19;86(7):1753–68. doi: 10.1158/0008-5472.CAN-25-1697 (PMC13044534; doi:10.1158/0008-5472.CAN-25-1697)
Supplement: Supplemental Figure 4 — Mass cytometry gating strategy of CD19+ cell subpopulations [file can-25-1697_supplemental_figure_4_suppsf4.pdf]

Supplemental Figure 4

Continued from Figure S1B

A

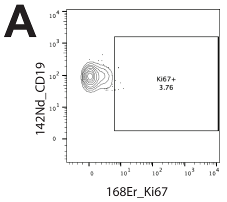

B

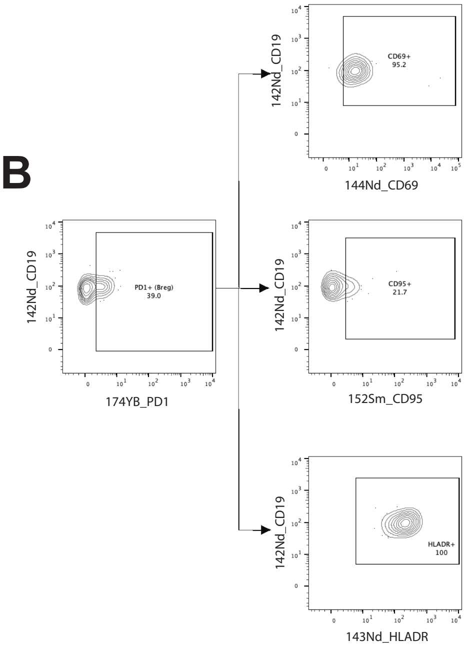

C

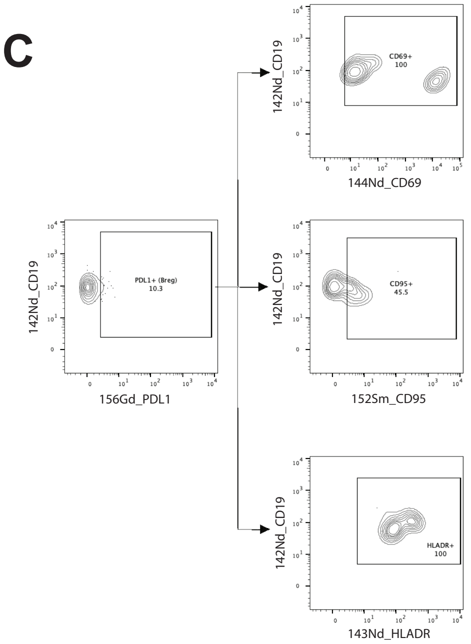

D

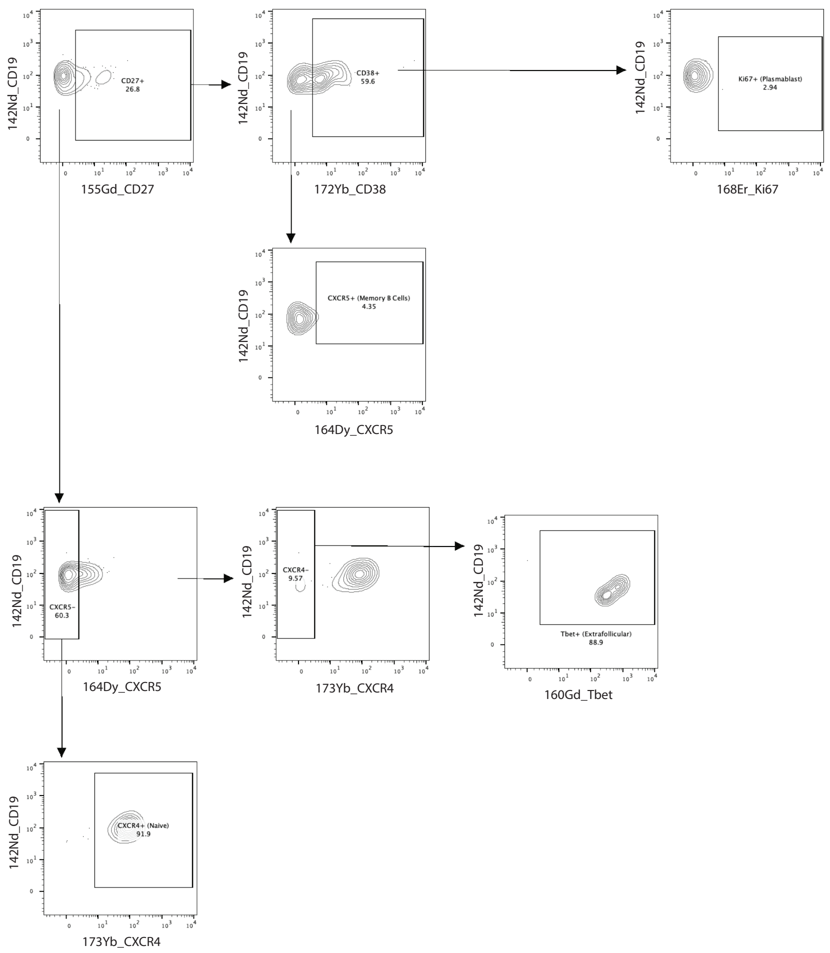

Supplemental Figure 4 Mass cytometry gating strategy of $CD19^+$ cell subpopulations. Representative plots show (A) Ki-67^+ cells, (B) PD-1^+ Breg, and $CD69^+$, $CD95^+$ and HLA-DR^+ subsets, (C) PD-$L1^+$ Breg and $CD69^+$, $CD95^+$ and HLA-DR^+ subsets, and (D) $CD27^+CD38^+$Ki-67^+ plasmablasts; $CD27^+CD38^-CXCR5^+$ memory B cells, $CD27^-CXCR5^+CXCR4^-$T-bet^+ extrafollicular B cells, and $CD27^-CXCR5^-CXCR4^+$ naïve B cells. Representative sample: P7. Bregs, B regulatory cells.
