# Supplementary material for: High-Dimensional Protein Analysis Uncovers Distinct Immunologic and Stromal Features in Primary and Metastatic Pancreatic Ductal Adenocarcinoma
Source: Cancer Res. 2025 Dec 19;86(7):1753–68. doi: 10.1158/0008-5472.CAN-25-1697 (PMC13044534; doi:10.1158/0008-5472.CAN-25-1697)
Supplement: Supplemental Figure 5 — Mass cytometry gating strategy of inhibitory immune checkpoint receptors present on CD4+ and CD8+ T cell populations [file can-25-1697_supplemental_figure_5_suppsf5.pdf]

Supplemental Figure 5

Continued from Figure S1B

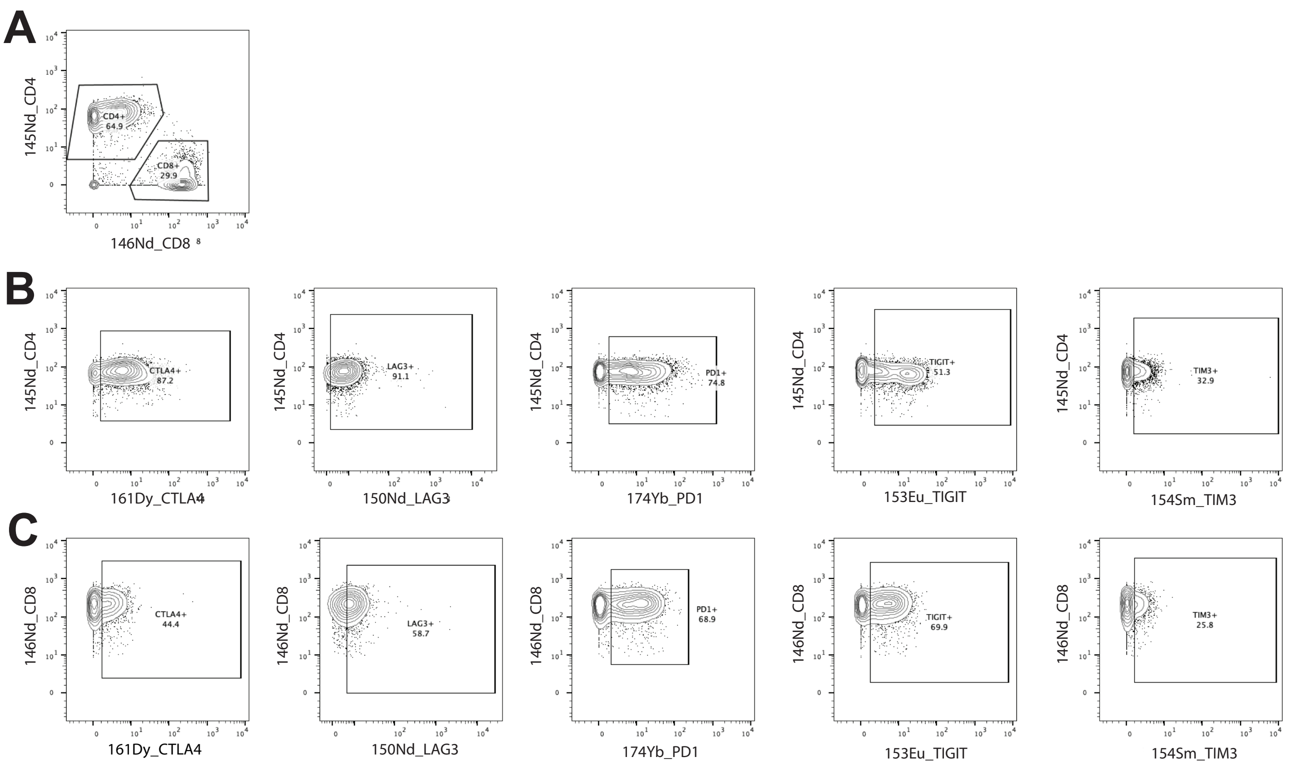

Supplemental Figure 5 Mass cytometry gating strategy of inhibitory immune checkpoint receptors present on $CD4^+$ and $CD8^+$ T cell populations. (A) $CD4^+$ and $CD8^+$ T cells gated from the $CD3^+$ population. Representative plots showing checkpoint molecules (CTLA-4, LAG-3, PD-1, TIGIT, and TIM-3) on (B) $CD4^+$ cells and (C) $CD8^+$ cells. Representative sample: P8.
