# Supplementary material for: High-Dimensional Protein Analysis Uncovers Distinct Immunologic and Stromal Features in Primary and Metastatic Pancreatic Ductal Adenocarcinoma
Source: Cancer Res. 2025 Dec 19;86(7):1753–68. doi: 10.1158/0008-5472.CAN-25-1697 (PMC13044534; doi:10.1158/0008-5472.CAN-25-1697)
Supplement: Supplemental Figure 6 — Single stain IHC validation of multiplex IHC panels [file can-25-1697_supplemental_figure_6_suppsf6.pdf]

Supplemental Figure 6

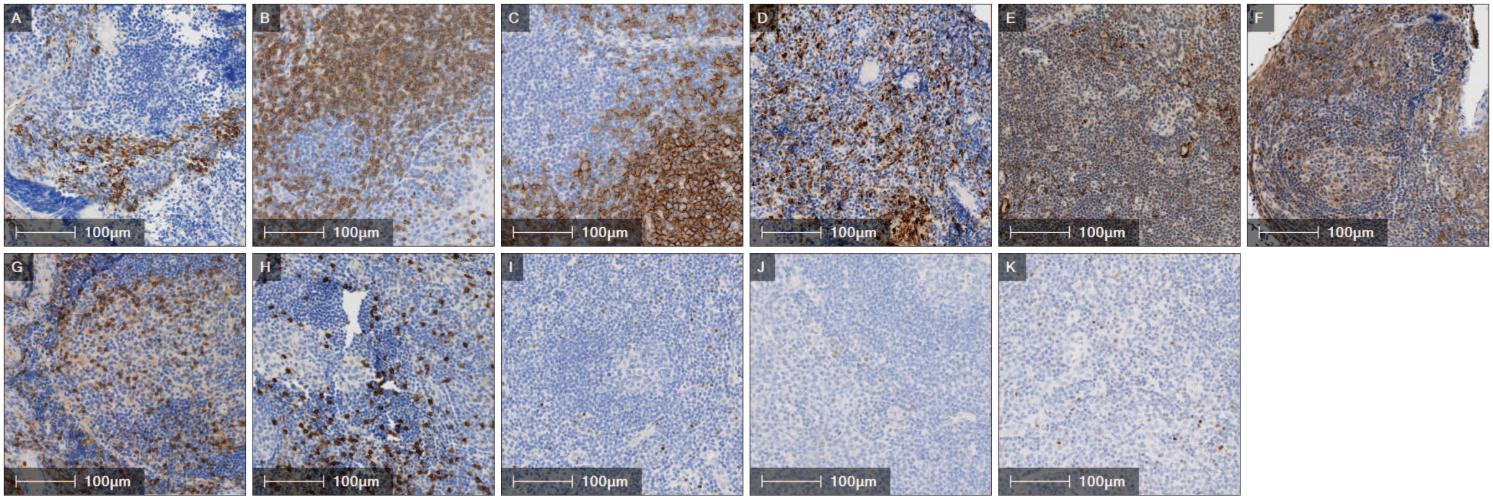

Supplemental Figure 6 Single stain IHC validation of multiplex IHC panels. Human tonsil tissue was used as a biologic control for validating single antibody staining prior to use on multiplex IHC staining panels. Representative images are provided for each stain: (A) CK19, (B) CD3, (C) CD19, (D) CD68, (E) α-SMA, (F) IL-6, (G) CD4, (H) CD8, (I) FoxP3, (J) RORγt and (K) T-bet. Scale bar: 100 μm.
