# Supplementary material for: High-Dimensional Protein Analysis Uncovers Distinct Immunologic and Stromal Features in Primary and Metastatic Pancreatic Ductal Adenocarcinoma
Source: Cancer Res. 2025 Dec 19;86(7):1753–68. doi: 10.1158/0008-5472.CAN-25-1697 (PMC13044534; doi:10.1158/0008-5472.CAN-25-1697)
Supplement: Supplemental Figure 7 — Density of phenotyped cells from mIHC analysis validates total cell quantification [file can-25-1697_supplemental_figure_7_suppsf7.pdf]

# Supplemental Figure 7

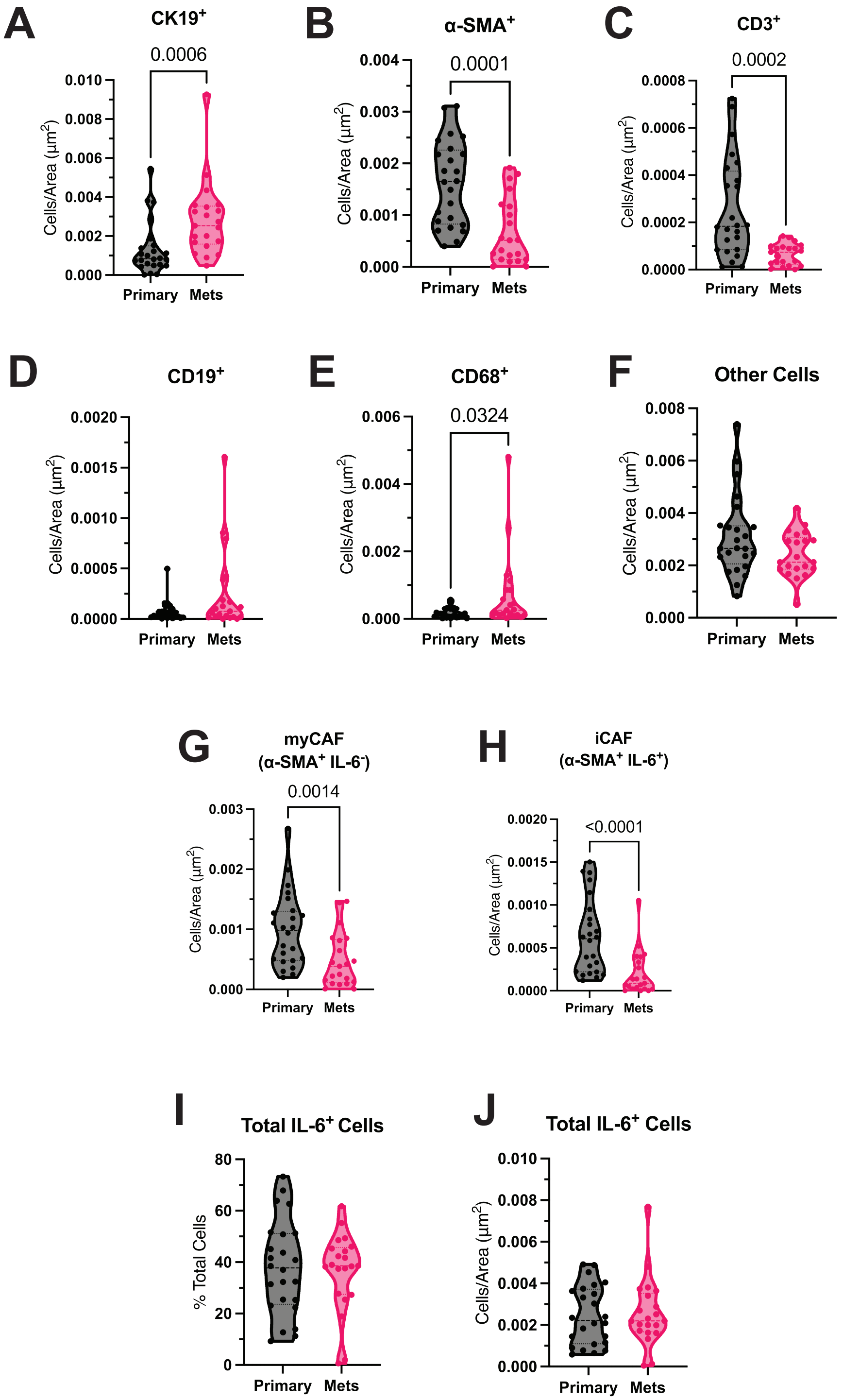

**Supplemental Figure 7** Density of phenotyped cells from mIHC analysis validates total cell quantification. Truncated violin plots show the abundance of (A) $CK19^+$, (B) α-$SMA^+$, (C) $CD3^+$, (D) $CD19^+$, (E) $CD68^+$, (F) other $DAPI^+$, (G) myCAF (α-$SMA^+$ IL-$6^-$), and (H) iCAF (α-$SMA^+$IL-$6^+$) cells in primary and metastatic tissue, normalized to tissue area ($\mu m^2$). Truncated violin plots also show total IL-$6^+$ cells as (I) a percentage of total cells and (J) density ($\mu m^2$) in primary and metastatic tissue. Statistical comparisons were done using Mann-Whitney tests, and significant p values are indicated. Sample size: n=24, primary; n=21, metastatic.
