# Supplementary material for: High-Dimensional Protein Analysis Uncovers Distinct Immunologic and Stromal Features in Primary and Metastatic Pancreatic Ductal Adenocarcinoma
Source: Cancer Res. 2025 Dec 19;86(7):1753–68. doi: 10.1158/0008-5472.CAN-25-1697 (PMC13044534; doi:10.1158/0008-5472.CAN-25-1697)
Supplement: Supplemental Figure 8 — Density of phenotyped cells from mIF analysis confirming total cell quantification [file can-25-1697_supplemental_figure_8_suppsf8.pdf]

Supplemental Figure 8

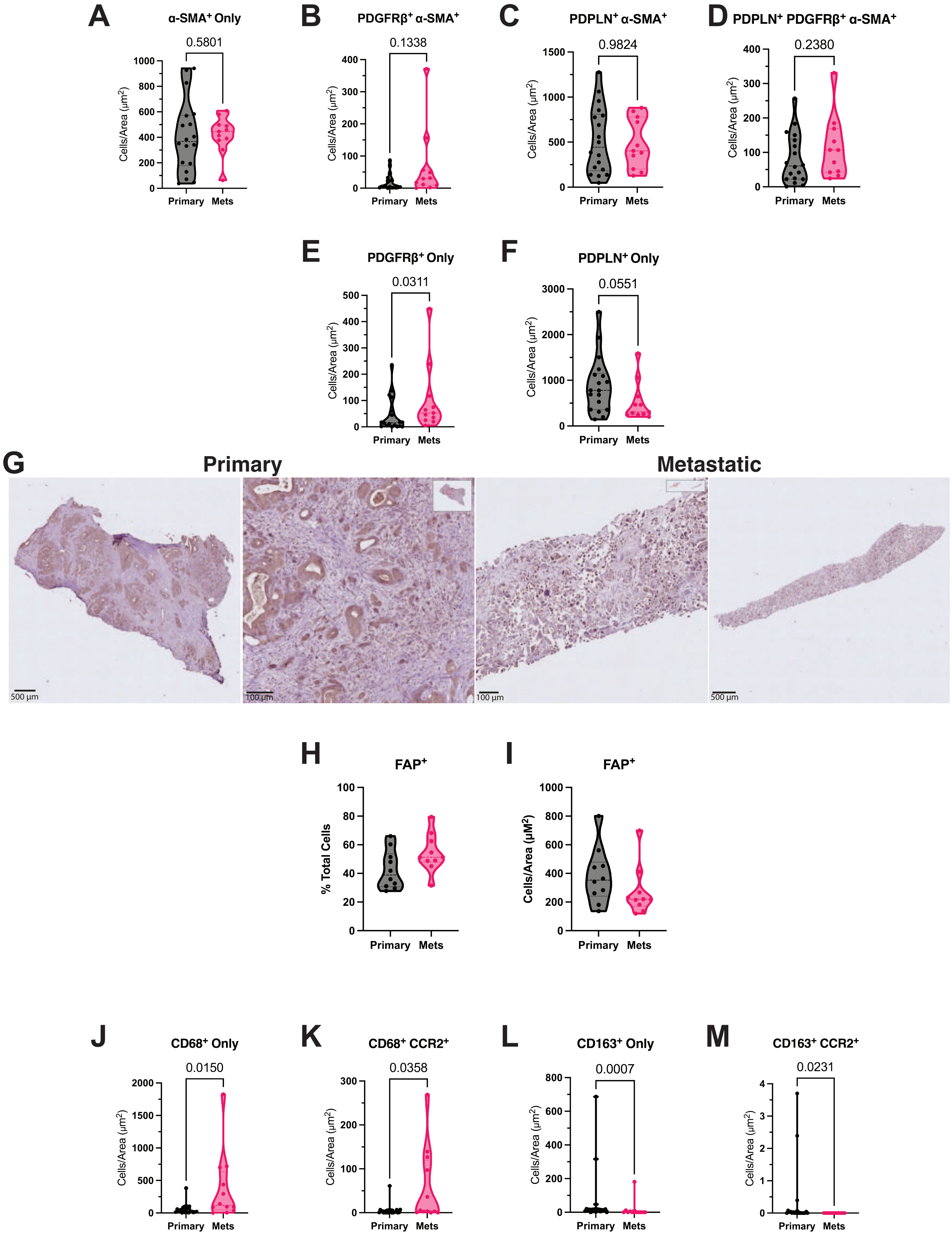

Supplemental Figure 8 Density of phenotyped cells from mIF analysis confirming total cell quantification. Truncated violin plots show the abundance of (A) α-SMA$^+$ only, (B) PDGFRβ$^+$ α-SMA$^+$, (C) PDPLN$^+$ α-SMA$^+$, (D) PDGFRβ$^+$ PDPLN$^+$ α-SMA$^+$, (E) PDFGRβ$^+$ only, (F) PDPLN$^+$ only, (G) Representative images of FAP IHC in PDAC tissues. Scale bar: outside panel, 500 μm; inside panel, 100 μm. Truncated violin plots show FAP$^+$ cell abundance as (H) percent of total cells and (I) density (um^2) in primary and metastatic tissue. (J) CD68$^+$, (K) CD68$^+$CCR2$^+$, (L) CD163$^+$, and (M) CD163$^+$CCR2$^+$ in primary and metastatic tissue, normalized to tissue area (μm^2). (K) Statistical comparisons were done using Mann-Whitney tests and significant p values are indicated. Sample size: fibroblast analysis, primary, n=18; metastatic, n=11; macrophage analysis, primary, n=20; metastatic, n=12; FAP analysis, primary, n=10; metastatic, n=10.
