# Supplementary material for: High-Dimensional Protein Analysis Uncovers Distinct Immunologic and Stromal Features in Primary and Metastatic Pancreatic Ductal Adenocarcinoma
Source: Cancer Res. 2025 Dec 19;86(7):1753–68. doi: 10.1158/0008-5472.CAN-25-1697 (PMC13044534; doi:10.1158/0008-5472.CAN-25-1697)
Supplement: Supplemental Figure 9 — Spatial analysis reveals distinct cellular distribution and proximity of immune cells to CK19+ cells in primary and metastatic PDAC tumors [file can-25-1697_supplemental_figure_9_suppsf9.pdf]

Supplemental Figure 9

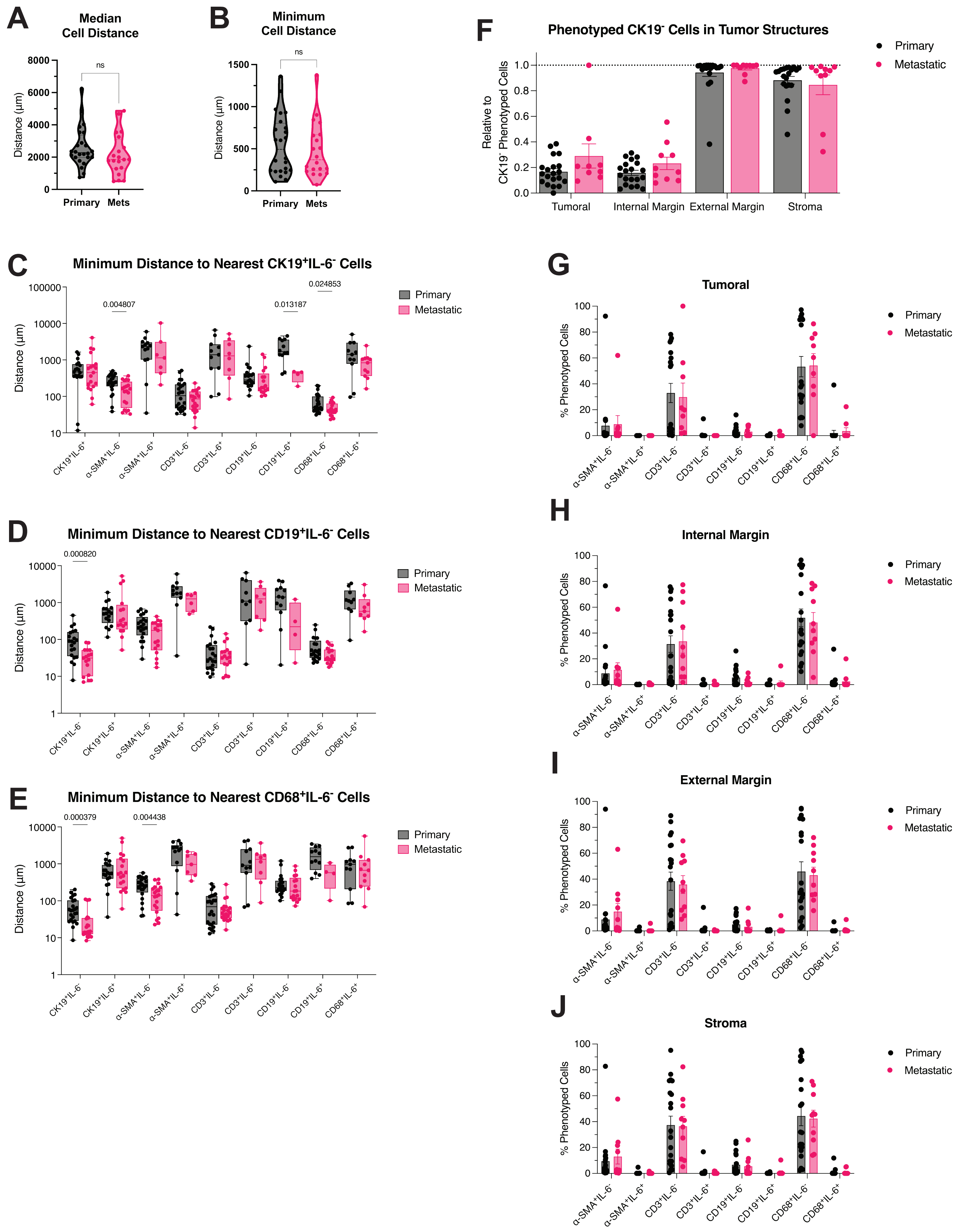

Supplemental Figure 9 Spatial analysis reveals distinct cellular distribution and proximity of immune cells to $CK19^+$ cells in primary and metastatic PDAC tumors. (A) Truncated violin plots of median cell distance, reflecting overall cell spacing across tissue samples (Mann-Whitney test, not significant). (B) Truncated violin plots of minimum cell distance, reflecting cell-to-cell interactions where proximity is critical for function (Mann-Whitney test, not significant). Box-and-whisker plots show minimum distances of (C) $CK19^+$, (D) $CD19^+$, and (E) $CD68^+$ cells (Mann-Whitney tests, significant p values indicated). (F) Bar graph quantifying the fraction of $CK19^-$ cells in four defined tumor structures (Two-Way ANOVA, not significant). (G-J) Bar graphs showing percent of each cell type in tumoral, internal margin, external margin, and stroma regions (Mann-Whitney test, not significant). Sample sizes: tissue spatial analysis: primary, n=24; metastatic, n=20; tumor structure analysis: primary, n=20; metastatic, n=10.
