# Supplementary material for: High-Dimensional Protein Analysis Uncovers Distinct Immunologic and Stromal Features in Primary and Metastatic Pancreatic Ductal Adenocarcinoma
Source: Cancer Res. 2025 Dec 19;86(7):1753–68. doi: 10.1158/0008-5472.CAN-25-1697 (PMC13044534; doi:10.1158/0008-5472.CAN-25-1697)
Supplement: Supplemental Figure 10 — Transcription factor levels in CD8+ T cells reveals trends toward suppressive phenotypes [file can-25-1697_supplemental_figure_10_suppsf10.pdf]

Supplemental Figure 10

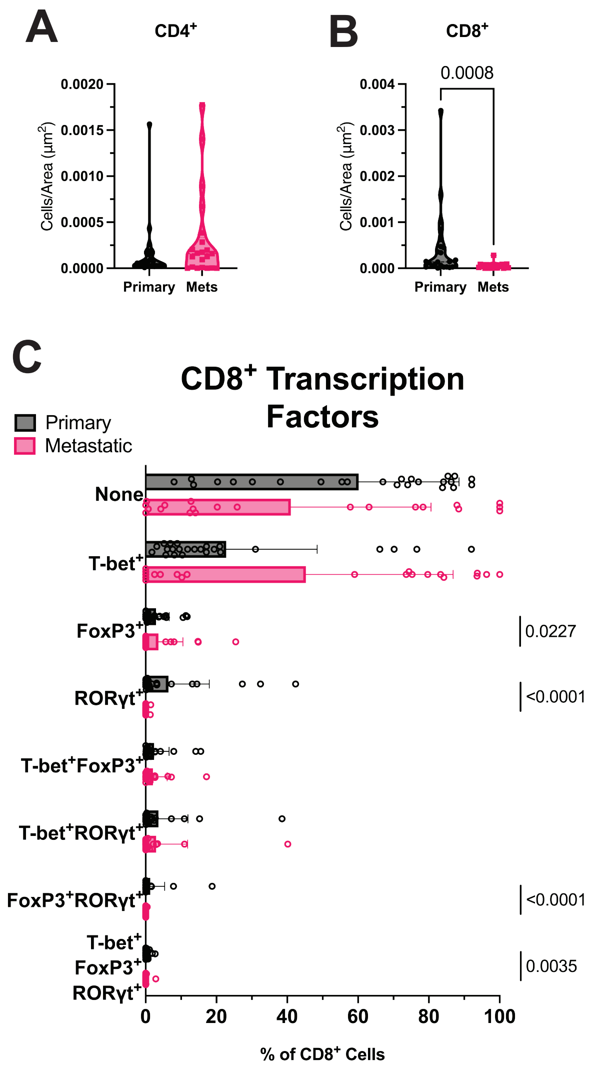

Supplemental Figure 10 Transcription factor levels in $CD8^+$ T cells reveals trends toward suppressive phenotypes. Truncated violin plots show the abundance of (A) $CD4^+$ and (B) $CD8^+$ in primary and metastatic tissue, relative to tissue area (μm^2). (C) Bar graph showing the percentage of transcription factor (T-bet, FoxP3 and RORγt) positive $CD8^+$ T cell populations, alone or in combination, represented as a proportion of total $CD8^+$ T cells. Statistical comparisons were done using Mann-Whitney tests; significant p values are indicated. Sample sizes: primary, n=24; metastatic, n=21.
