# Supplementary material for: High-Dimensional Protein Analysis Uncovers Distinct Immunologic and Stromal Features in Primary and Metastatic Pancreatic Ductal Adenocarcinoma
Source: Cancer Res. 2025 Dec 19;86(7):1753–68. doi: 10.1158/0008-5472.CAN-25-1697 (PMC13044534; doi:10.1158/0008-5472.CAN-25-1697)
Supplement: Supplemental Figure 11 — Mass Cytometry quantification of lymphocyte populations and proliferation in primary and metastatic tissue [file can-25-1697_supplemental_figure_11_suppsf11.pdf]

Supplemental Figure 11

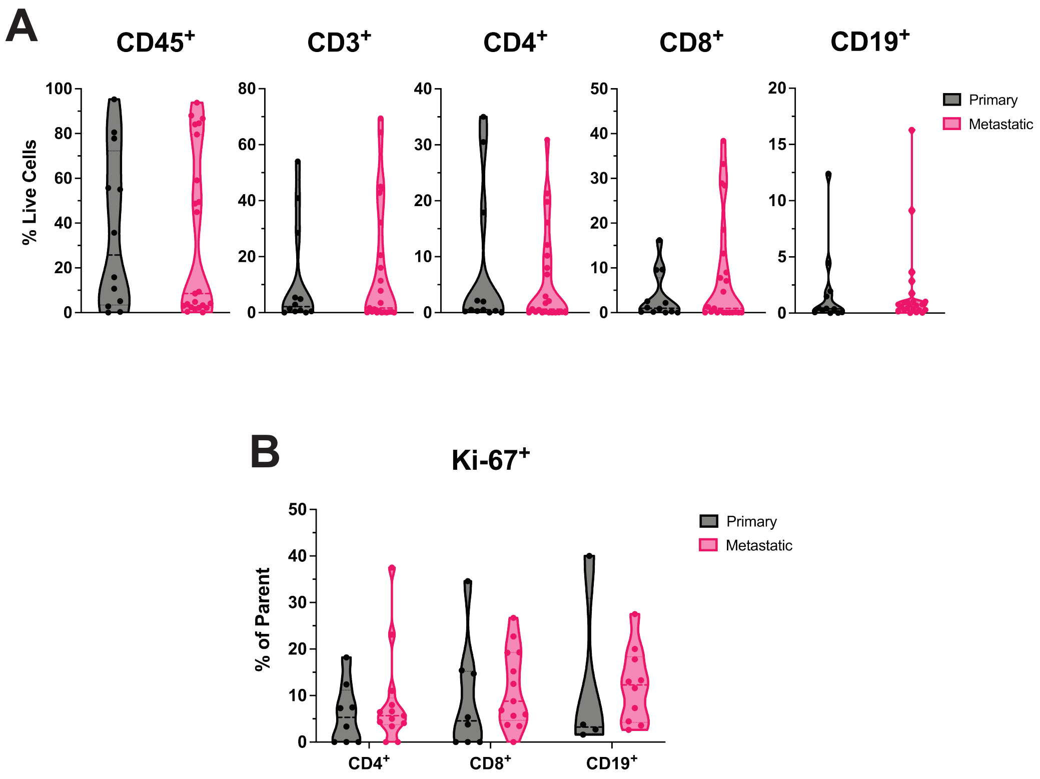

Supplemental Figure 11 Mass Cytometry quantification of lymphocyte populations and proliferation in primary and metastatic tissue. (A) Truncated violin plots showing the frequency of $CD45^+$, $CD3^+$, $CD4^+$, $CD8^+$, and $CD19^+$ lymphocyte populations as a percentage of live cells (Mann-Whitney test, not significant). Sample size: primary, n=12; metastatic, n=23. (B) Truncated violin plots showing Ki-67 expression on $CD4^+$, $CD8^+$ and $CD19^+$ cells as a percentage of total parent population (Mann-Whitney test, not significant). Sample size: $CD4^+$ and $CD8^+$, primary, n=8; metastatic, n=13. $CD19^+$, primary, n=4; metastatic, n=10.
