# Supplementary material for: High-Dimensional Protein Analysis Uncovers Distinct Immunologic and Stromal Features in Primary and Metastatic Pancreatic Ductal Adenocarcinoma
Source: Cancer Res. 2025 Dec 19;86(7):1753–68. doi: 10.1158/0008-5472.CAN-25-1697 (PMC13044534; doi:10.1158/0008-5472.CAN-25-1697)
Supplement: Supplemental Figure 12 — Mass cytometry analysis of myeloid and NK cells in primary and metastatic PDAC [file can-25-1697_supplemental_figure_12_suppsf12.pdf]

# Supplemental Figure 12

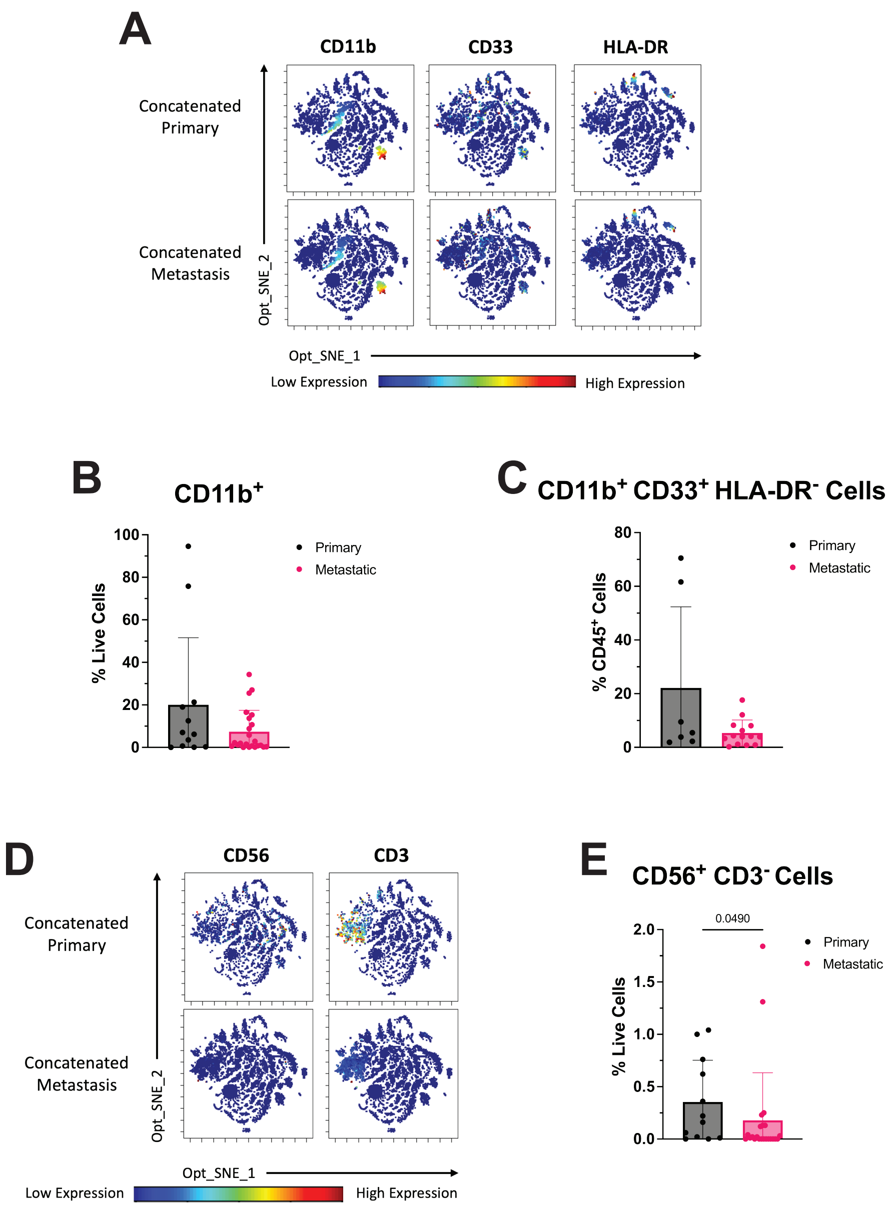

**Supplemental Figure 12** Mass cytometry analysis of myeloid and NK cells in primary and metastatic PDAC. (A) Opt_SNE plots showing levels of CD11b, CD33, and HLA-DR in concatenated primary (top) and metastatic (bottom) samples. (B) Quantification of $CD11b^+$ cells as a percentage of live cells. (C) Quantification of $CD11b^+$ $CD33^+$ $HLA\text{-}DR^-$ cells as a percentage of $CD45^+$ cells (Mann-Whitney test, not significant). (D) Opt_SNE plots showing expression of CD56 and CD3 in concatenated primary (top) and metastatic (bottom) samples. (E) Quantification of $CD56^+$ $CD3^-$ NK cells as a percentage of live cells (Mann-Whitney test, p value indicated). Sample sizes: myeloid cell analysis (≥25 $CD11b^+$ cells), primary, n=7; metastatic, n=14. $CD11b^+$ and $CD56^+CD3^-$ analysis, primary, n=12; metastatic, n=23.
