# Supplementary material for: High-Dimensional Protein Analysis Uncovers Distinct Immunologic and Stromal Features in Primary and Metastatic Pancreatic Ductal Adenocarcinoma
Source: Cancer Res. 2025 Dec 19;86(7):1753–68. doi: 10.1158/0008-5472.CAN-25-1697 (PMC13044534; doi:10.1158/0008-5472.CAN-25-1697)
Supplement: Supplemental Figure 13 — Mass cytometry analysis of T cell subsets in primary and metastatic PDAC [file can-25-1697_supplemental_figure_13_suppsf13.pdf]

Supplemental Figure 13

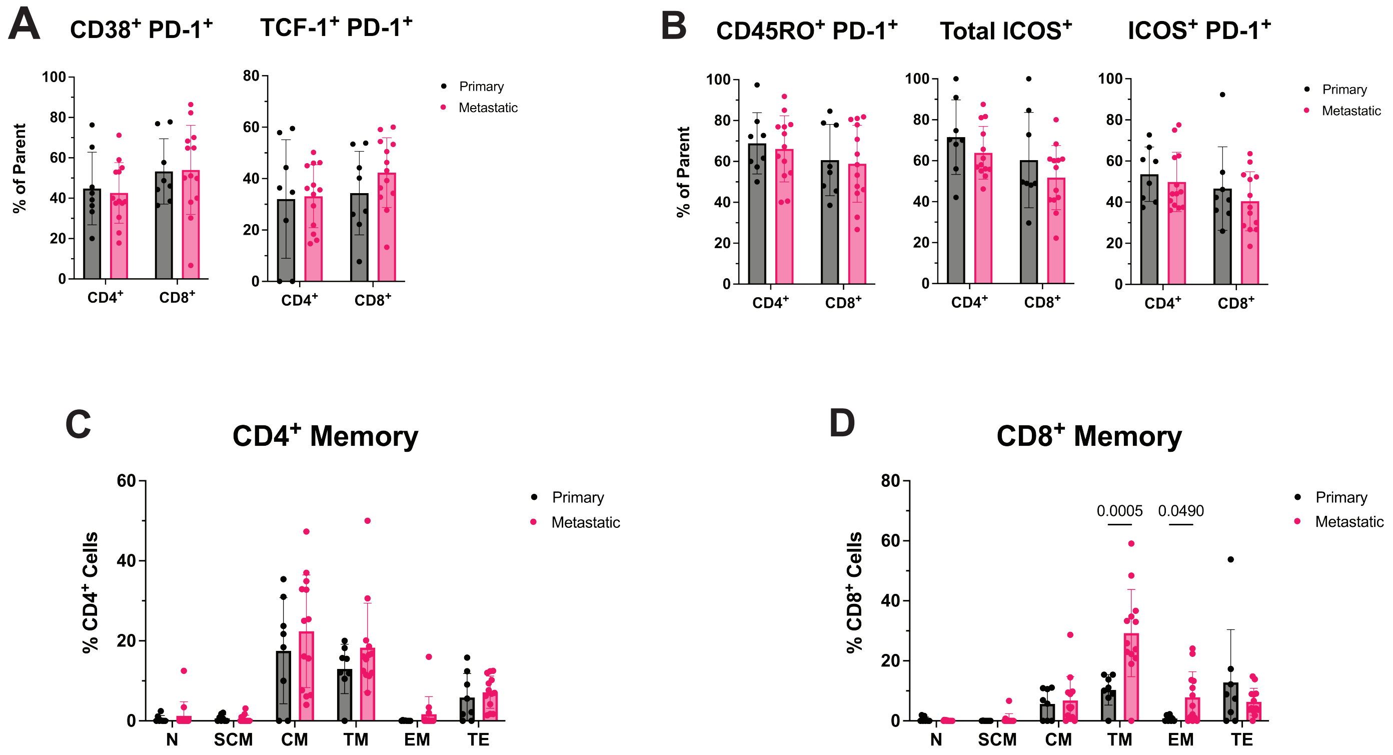

Supplemental Figure 13 Mass cytometry analysis of T cell subsets in primary and metastatic PDAC. Bar graphs showing $CD4^+$ and $CD8^+$ T cells expressing (A) $CD38^+PD\text{-}1^+$ or $TCF\text{-}1^+PD\text{-}1^+$ and (B) $CD45RO^+$ $PD\text{-}1^+$, total $ICOS^+$, and $ICOS^+PD\text{-}1^+$. Memory T cell subsets are shown for (C) $CD4^+$ and (D) $CD8^+$ T cells (N, SCM, CM, TM, EM, TE) (Mann-Whitney tests, significant p values are indicated). Sample sizes: $CD4^+$ and $CD8^+$, primary, n=8; metastatic, n=13. N, naïve ($CD45RO^-CD127^+CD27^+CCR7^+CD95^-$); SCM, stem-like central memory ($CD45RO^-CD127^+CD27^+CCR7^+CD95^+$); CM, central memory ($CD45RO^+CD127^+CD27^+CCR7^+CD95^+$); TM, transitional memory ($CD45RO^+CCR7^-CD27^+CD95^+$); EM, effector memory ($CD45RO^-CD127^-CD27^-CD95^+$); TE, terminal effector ($CD45RO^+CCR7^-CD27^-CD95^+CD127^-$).
