# Supplementary material for: High-Dimensional Protein Analysis Uncovers Distinct Immunologic and Stromal Features in Primary and Metastatic Pancreatic Ductal Adenocarcinoma
Source: Cancer Res. 2025 Dec 19;86(7):1753–68. doi: 10.1158/0008-5472.CAN-25-1697 (PMC13044534; doi:10.1158/0008-5472.CAN-25-1697)
Supplement: Supplemental Figure 14 — Mass cytometry analysis of B cell subsets and their activation markers in PDAC [file can-25-1697_supplemental_figure_14_suppsf14.pdf]

Supplemental Figure 14

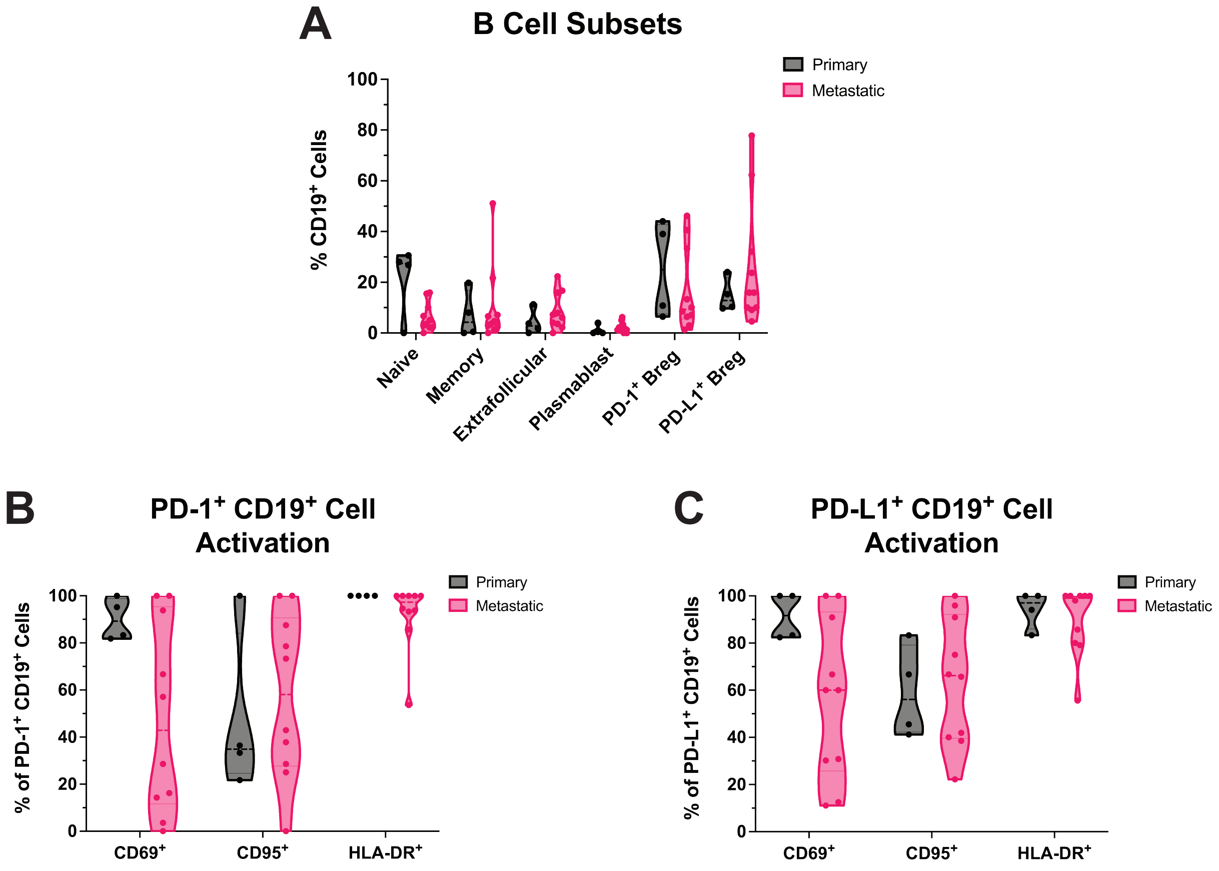

Supplemental Figure 14 Mass cytometry analysis of B cell subsets and their activation markers in PDAC. (A) Truncated violin plots showing naïve, memory, extrafollicular, plasmablast, PD-1$^+$, and PD-L1$^+$ B cell subsets as a percentage of CD19$^+$ cells (Mann-Whitney tests, not significant). Truncated violin plots showing (B) CD69$^+$, CD95$^+$ and HLA-DR$^+$ of PD-1$^+$CD19$^+$ cells and (C) PD-L1$^+$ CD19$^+$ cells, represented as a percentage of parent population (Mann-Whitney tests, not significant). Sample sizes: CD19$^+$ cells: primary, n=4; metastatic, n=10.
