# Supplementary material for: High-Dimensional Protein Analysis Uncovers Distinct Immunologic and Stromal Features in Primary and Metastatic Pancreatic Ductal Adenocarcinoma
Source: Cancer Res. 2025 Dec 19;86(7):1753–68. doi: 10.1158/0008-5472.CAN-25-1697 (PMC13044534; doi:10.1158/0008-5472.CAN-25-1697)
Supplement: Supplemental Figure 15 — Opt_SNE plots of checkpoint marker expression on CD4+ and CD8+ T cells [file can-25-1697_supplemental_figure_15_suppsf15.pdf]

Supplemental Figure 15

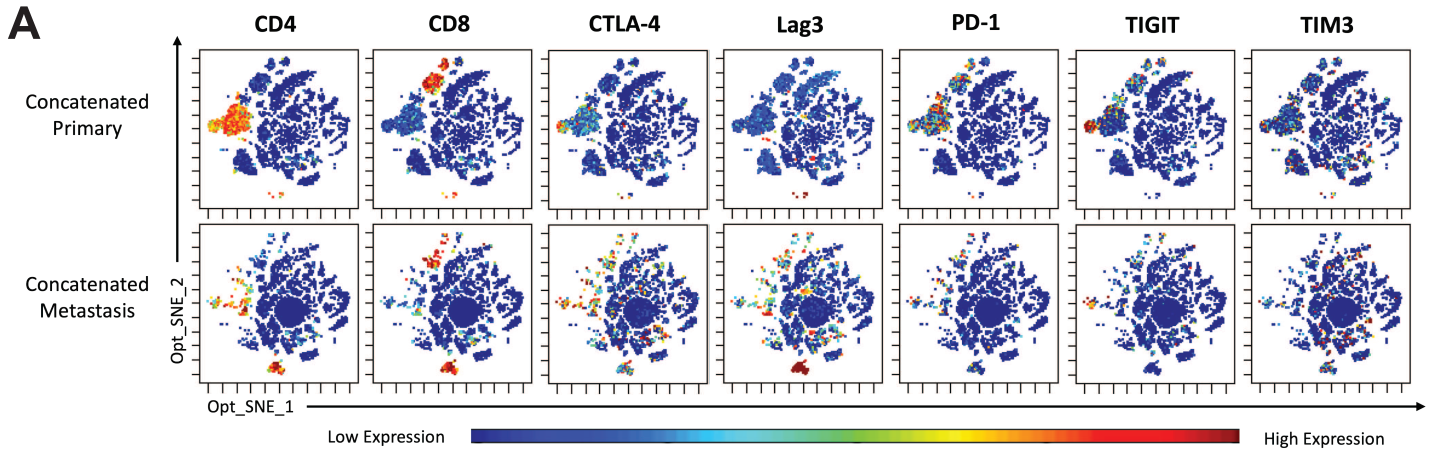

Supplemental Figure 15 Opt_SNE plots of checkpoint marker expression on $CD4^+$ and $CD8^+$ T cells. (A) Expression of CTLA-4, LAG-3, PD-1, TIGIT and TIM-3, along with CD4 and CD8, displayed on unsupervised Opt_SNE plots of live single cells in concatenated primary (top) and metastatic (bottom) samples. Opt_SNE, optimizes t-distributed stochastic neighbor embedding.
