# Supplementary material for: High-Dimensional Protein Analysis Uncovers Distinct Immunologic and Stromal Features in Primary and Metastatic Pancreatic Ductal Adenocarcinoma
Source: Cancer Res. 2025 Dec 19;86(7):1753–68. doi: 10.1158/0008-5472.CAN-25-1697 (PMC13044534; doi:10.1158/0008-5472.CAN-25-1697)
Supplement: Supplemental Figure 16 — Matched primary and metastatic PDAC samples validates trends observed in the larger cohort analysis [file can-25-1697_supplemental_figure_16_suppsf16.pdf]

Supplemental Figure 16

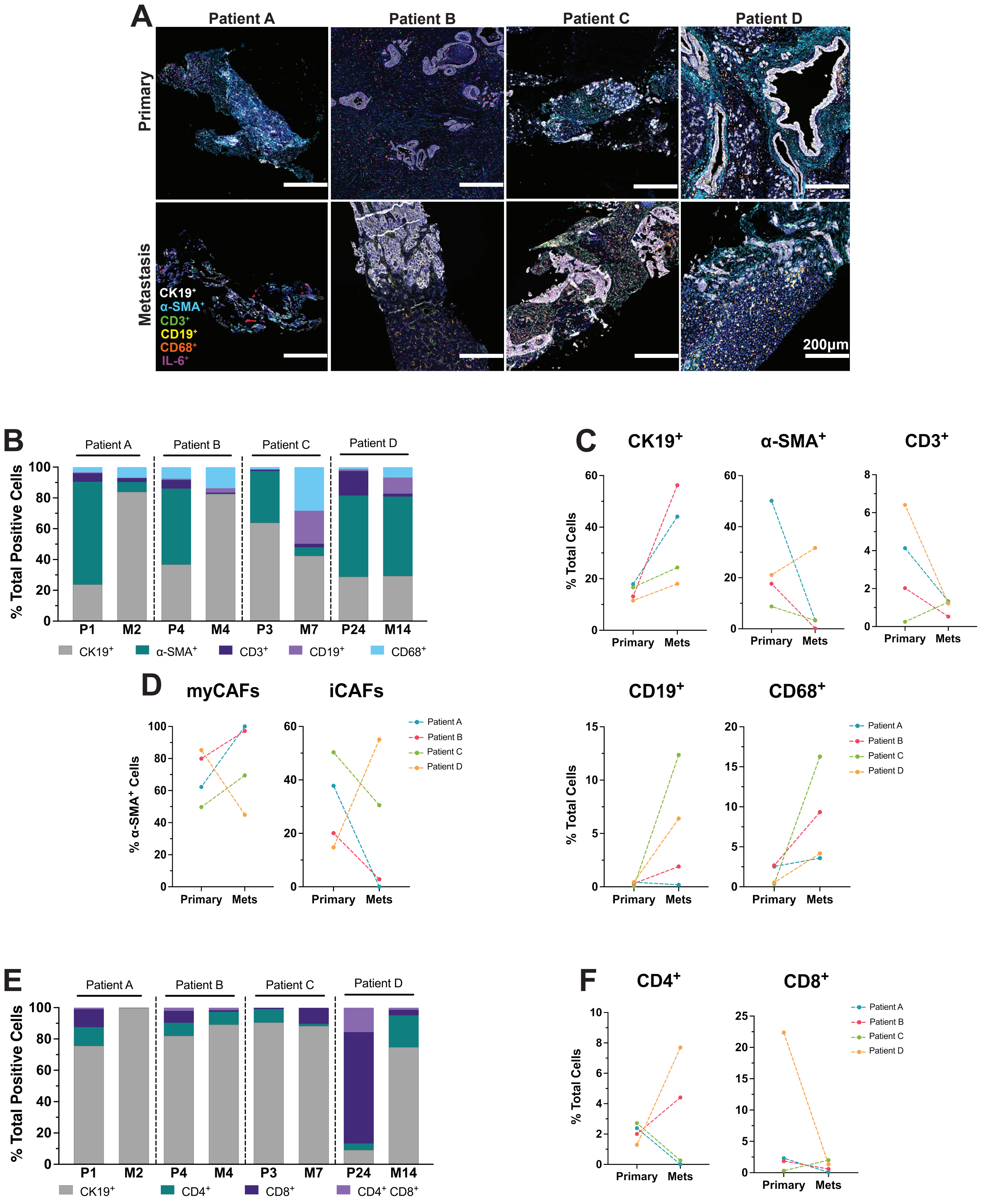

Supplemental Figure 16 Matched primary and metastatic PDAC samples validates trends observed in the larger cohort analysis. (A) Representative images of three primary (top) and patient-matched metastatic (bottom) tumor samples. Antibody panel: CK19 (white), α-SMA (cyan), CD3 (green), CD19 (yellow), CD68 (red), IL-6 (purple). Scale bar: 200 μm. (B) Stacked bar graph showing the distribution of $CK19^+$, $\alpha\text{-SMA}^+$, $CD3^+$, $CD19^+$, and $CD68^+$ cells for each patient sample. (C) Line plots comparing $CK19^+$, $\alpha\text{-SMA}^+$, $CD3^+$, $CD19^+$, and $CD68^+$ cell frequency between primary and metastatic tumors. (D) Lines plots comparing myCAF and iCAF subtypes between matched tumors (Wilcoxon test, not significant). (E) Stacked bar graph showing the distribution of $CK19^+$, $CD4^+$, $CD8^+$, and $CD4^+CD8^+$ cells for each patient. (F) Line plots comparing $CD4^+$ and $CD8^+$ T cells between primary and metastatic tumors (Wilcoxon test, not significant). n=4 matched patient samples.
