# Supplementary figures and images for: High-Dimensional Protein Analysis Uncovers Distinct Immunologic and Stromal Features in Primary and Metastatic Pancreatic Ductal Adenocarcinoma
Source: Cancer Res. 2025 Dec 19;86(7):1753–68. doi: 10.1158/0008-5472.CAN-25-1697 (PMC13044534; doi:10.1158/0008-5472.CAN-25-1697)

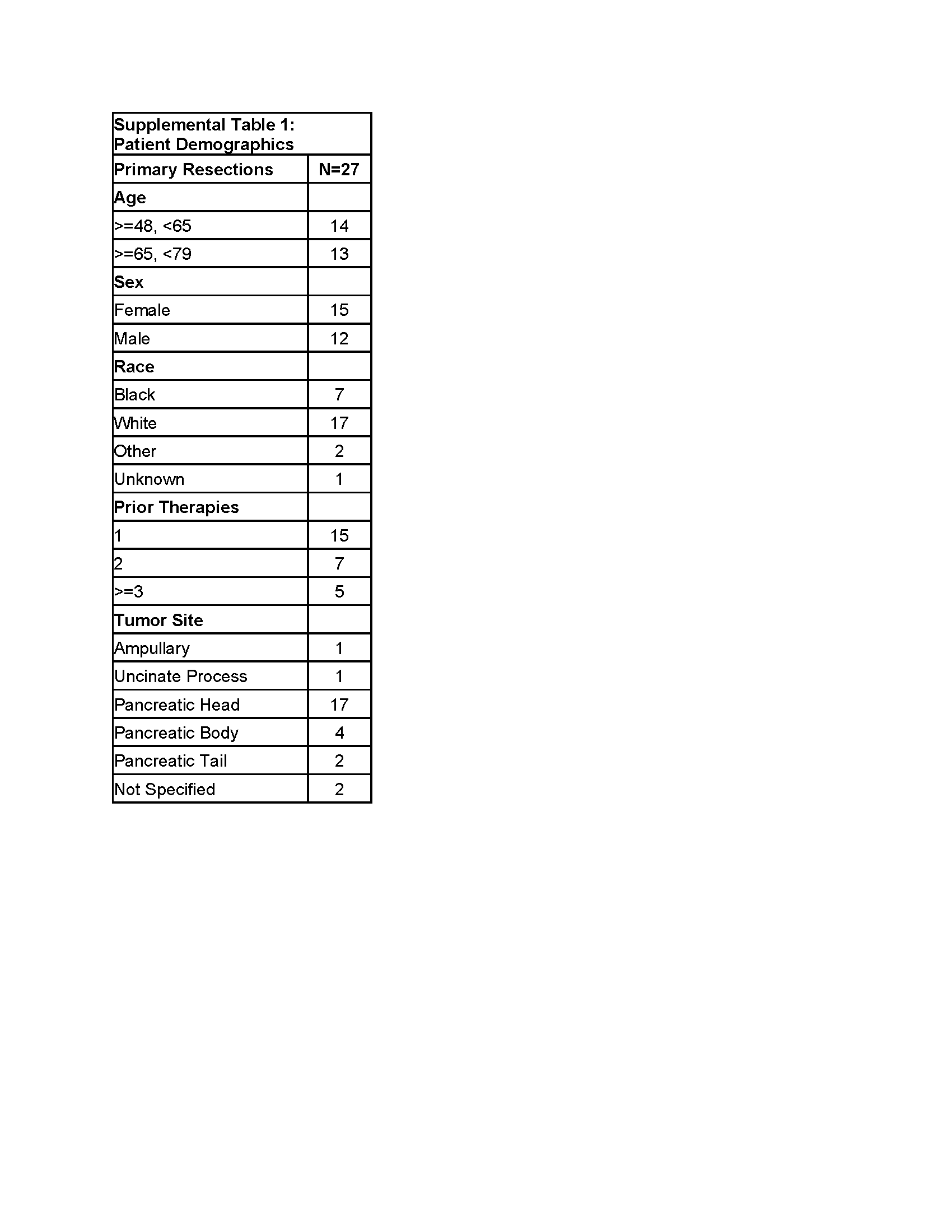

Supplement: Supplemental Table 1 — Demographics of patients with primary tumors [file can-25-1697_supplemental_table_1_suppst1.png]

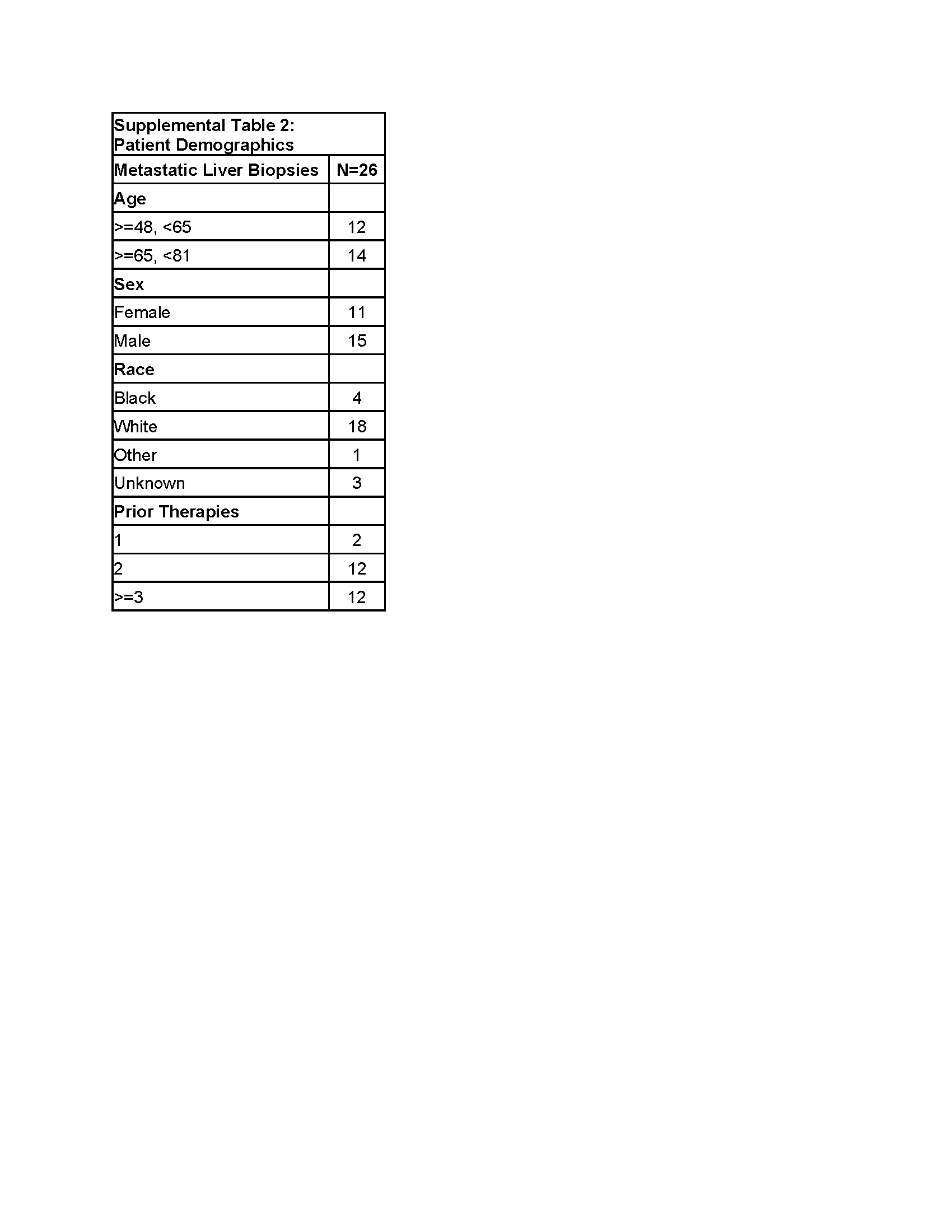

Supplement: Supplemental Table 2 — Demographics of metastatic patients [file can-25-1697_supplemental_table_2_suppst2.png]

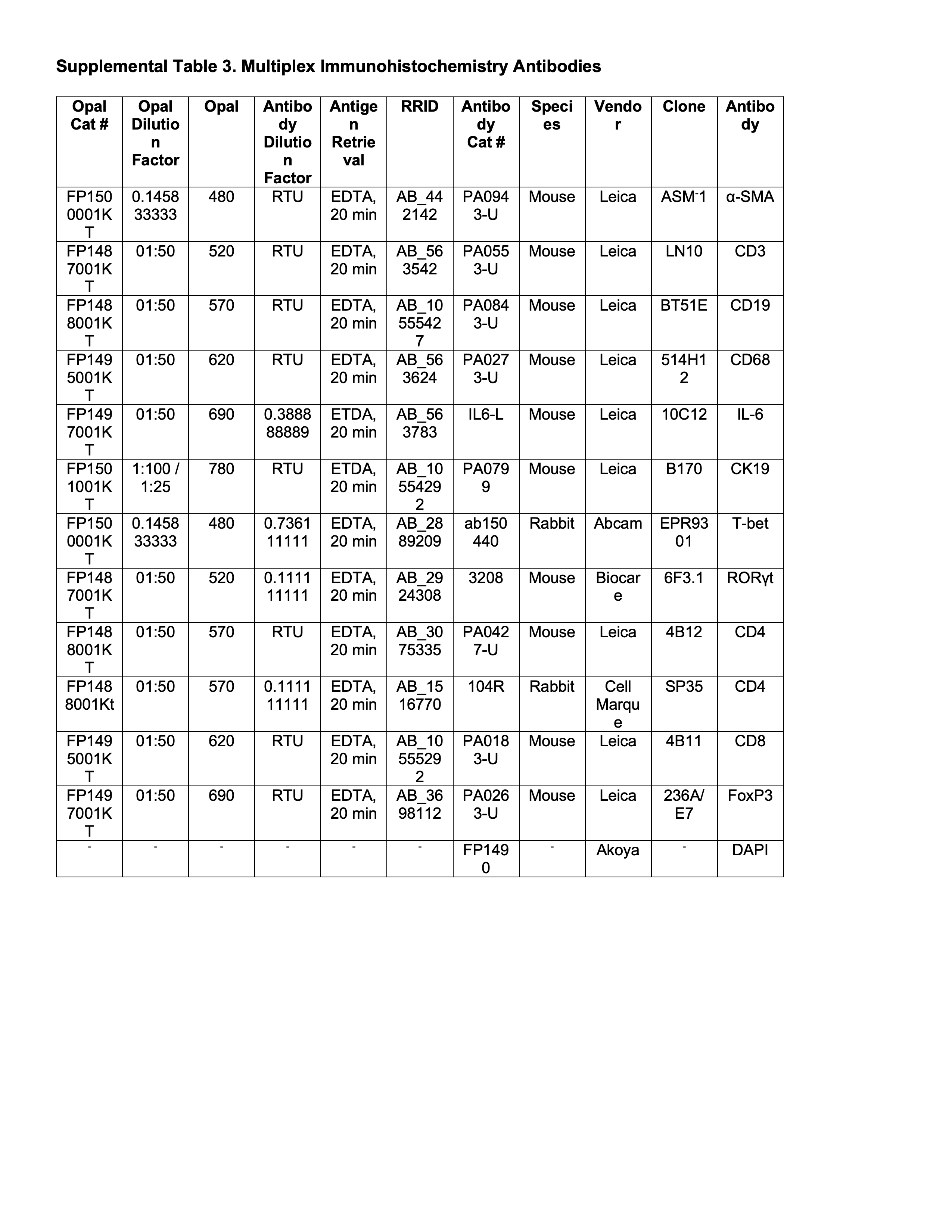

Supplement: Supplemental Table 3 — Multiplex IHC antibodies [file can-25-1697_supplemental_table_3_suppst3.png]

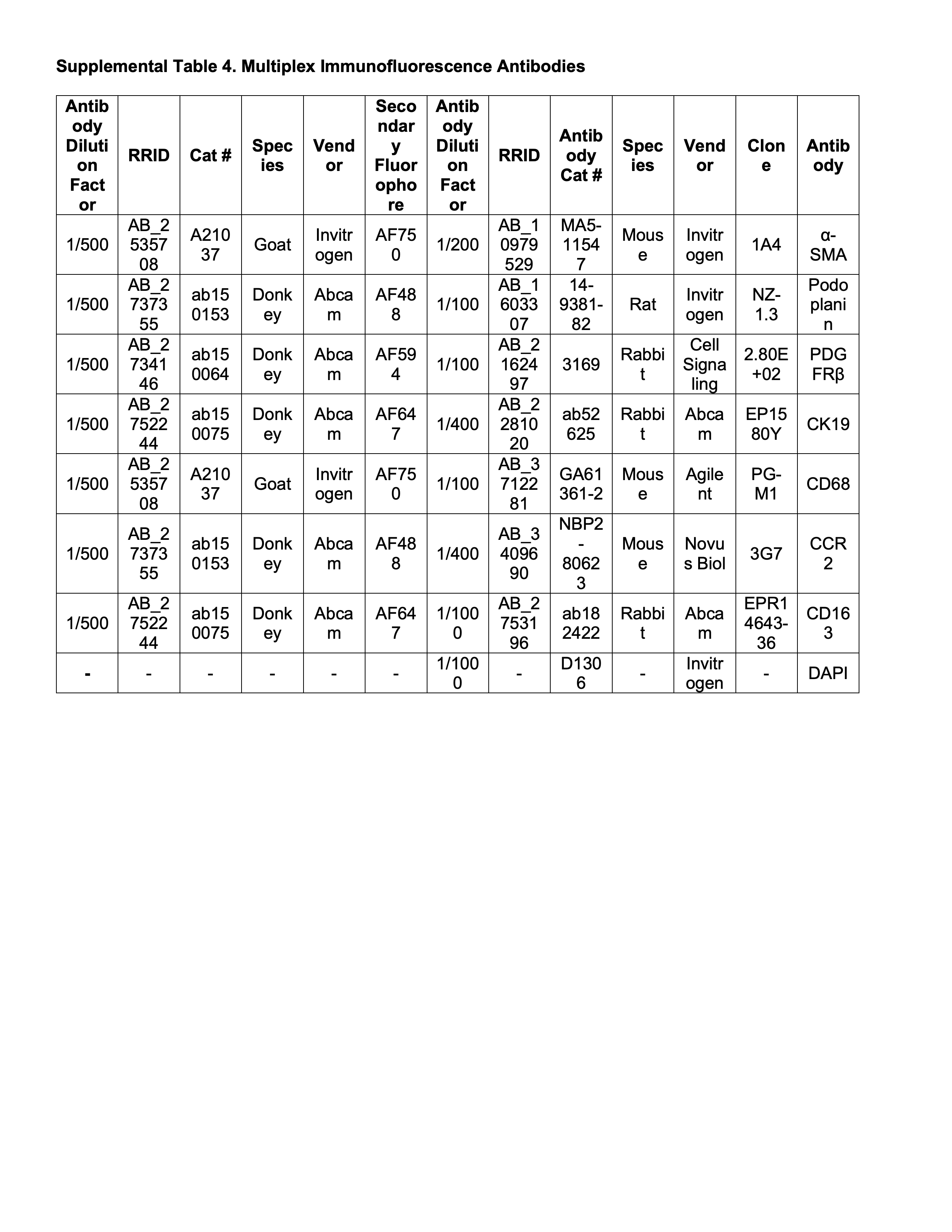

Supplement: Supplemental Table 4 — Additional multiplex IHC antibodies [file can-25-1697_supplemental_table_4_suppst4.png]

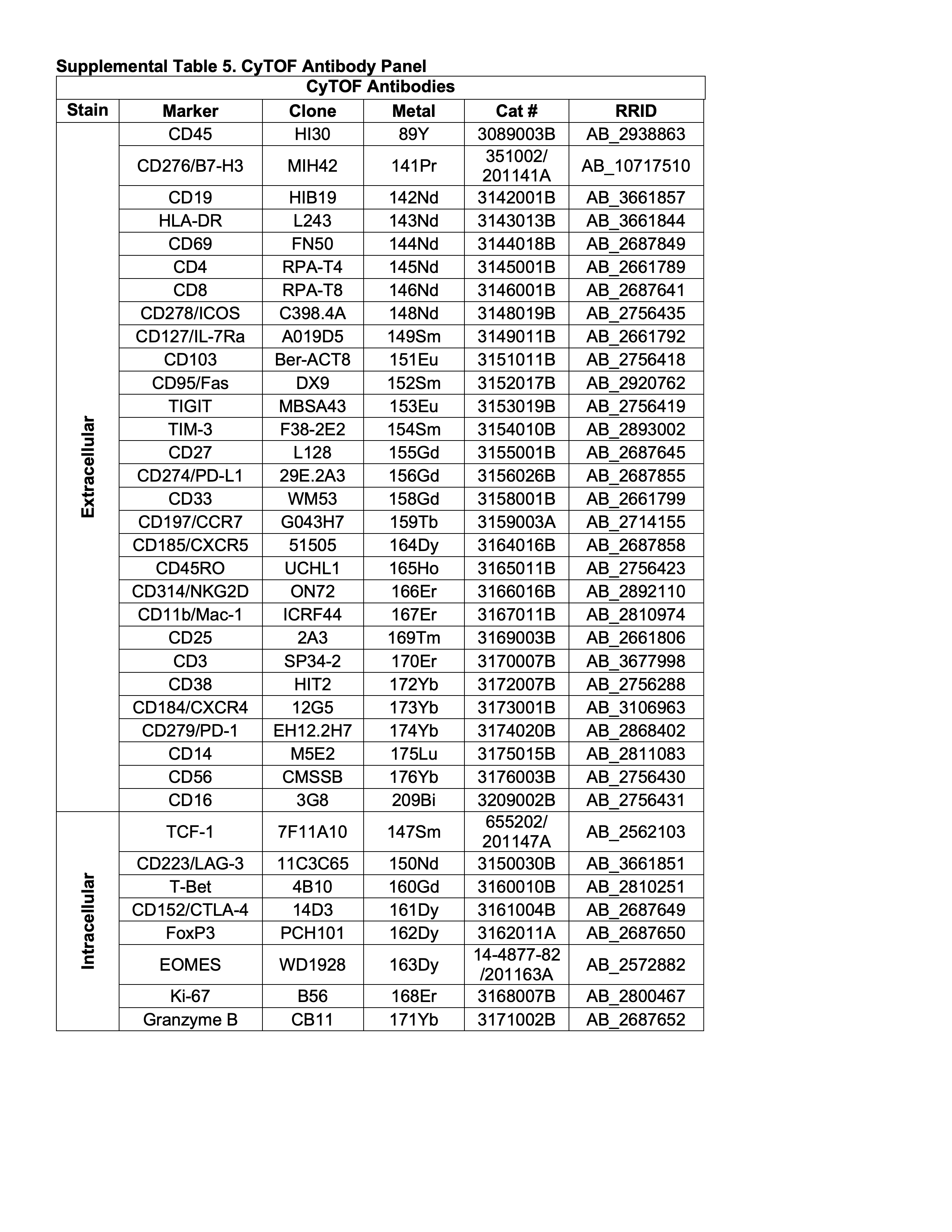

Supplement: Supplemental Table 5 — CyTOF antibody panel [file can-25-1697_supplemental_table_5_suppst5.png]

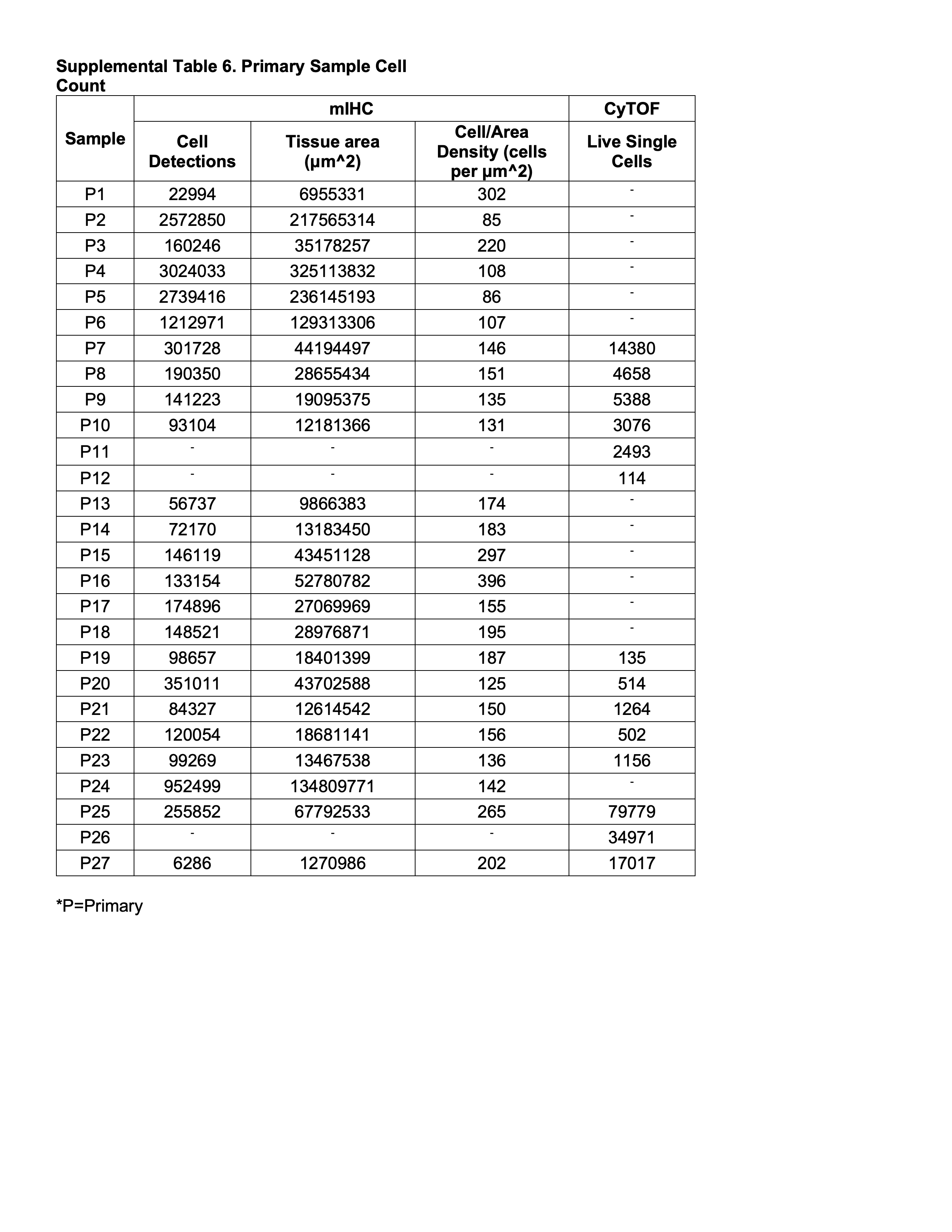

Supplement: Supplemental Table 6 — Primary sample cell count [file can-25-1697_supplemental_table_6_suppst6.png]

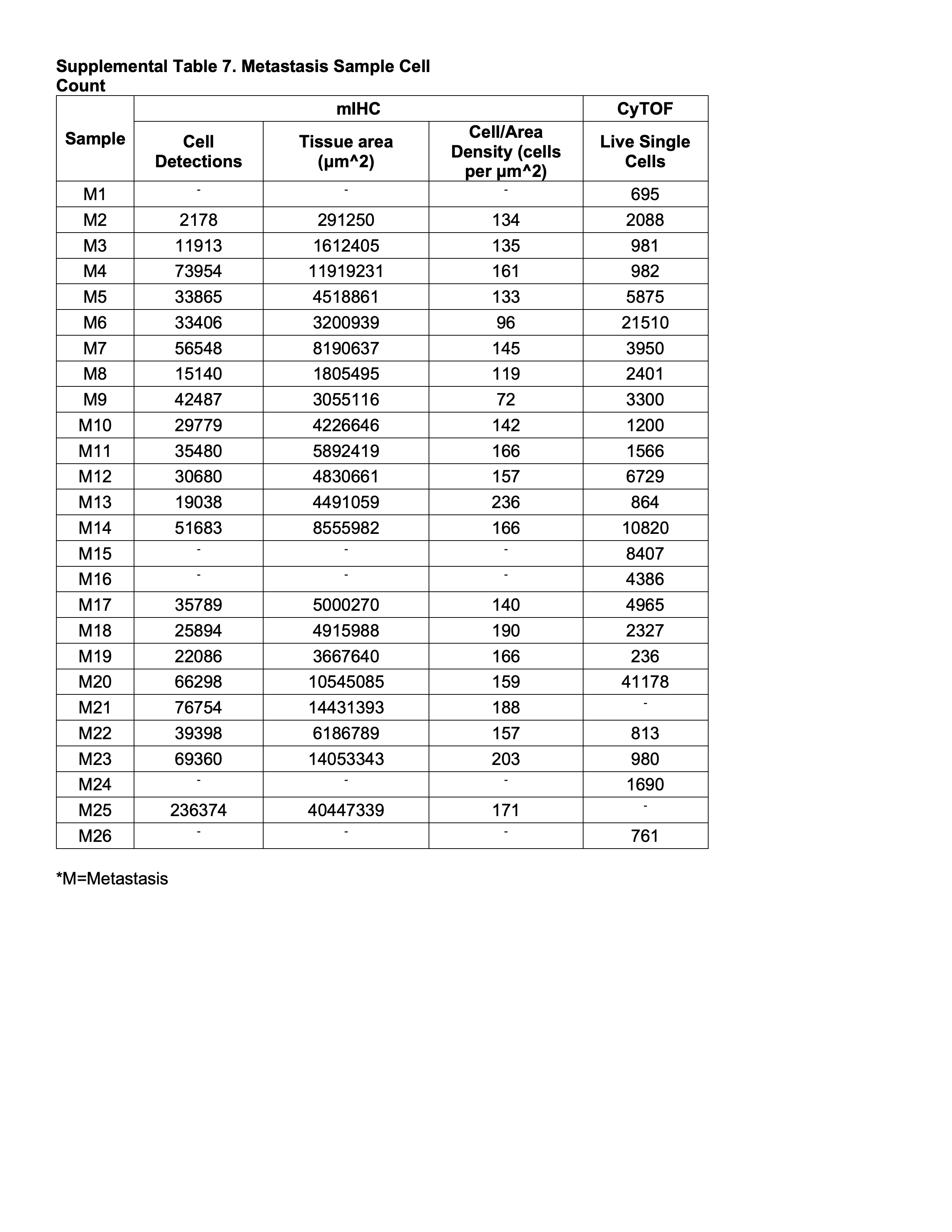

Supplement: Supplemental Table 7 — Metastasis sample cell count [file can-25-1697_supplemental_table_7_suppst7.png]

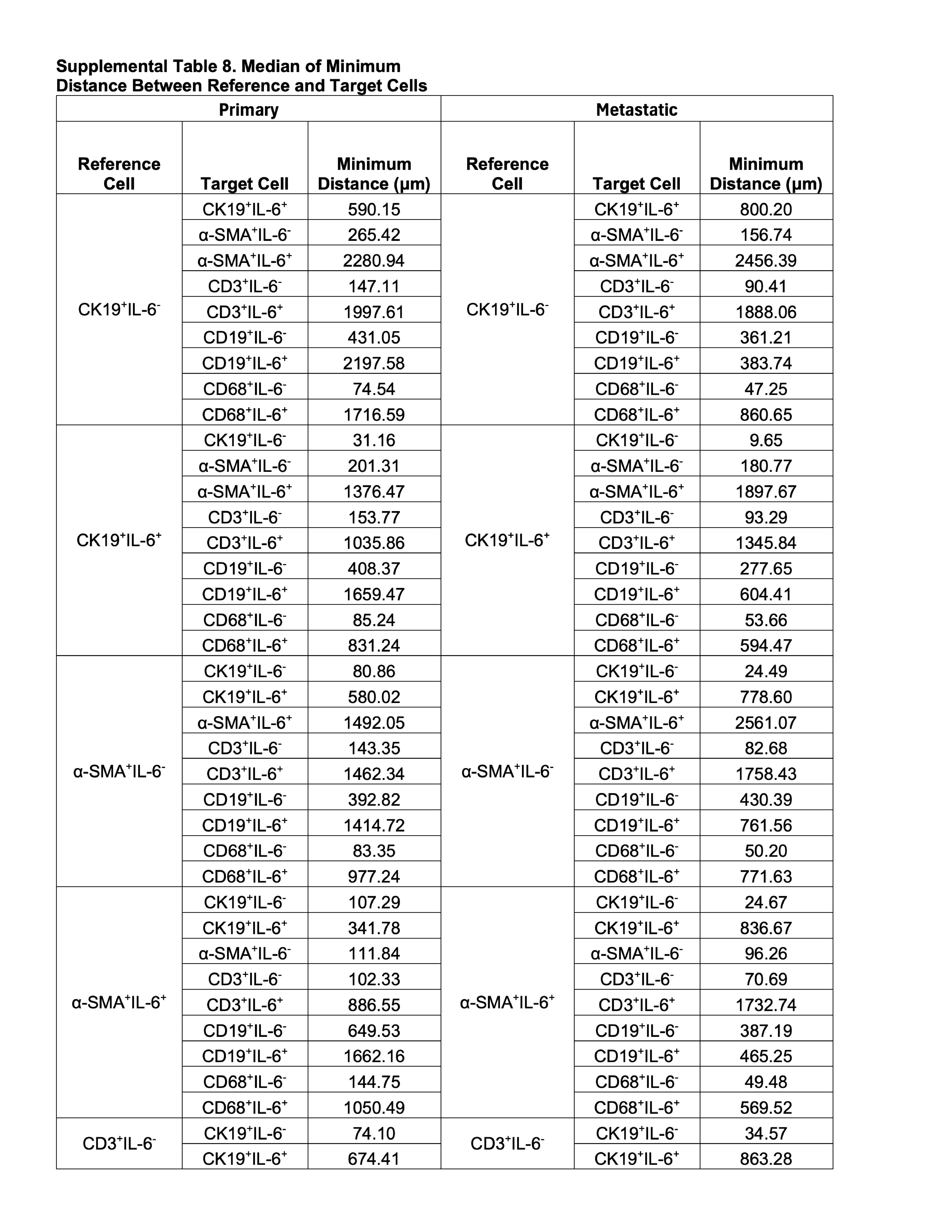

Supplement: Supplemental Table 8 — Distance between cells [file can-25-1697_supplemental_table_8_suppst8.png]

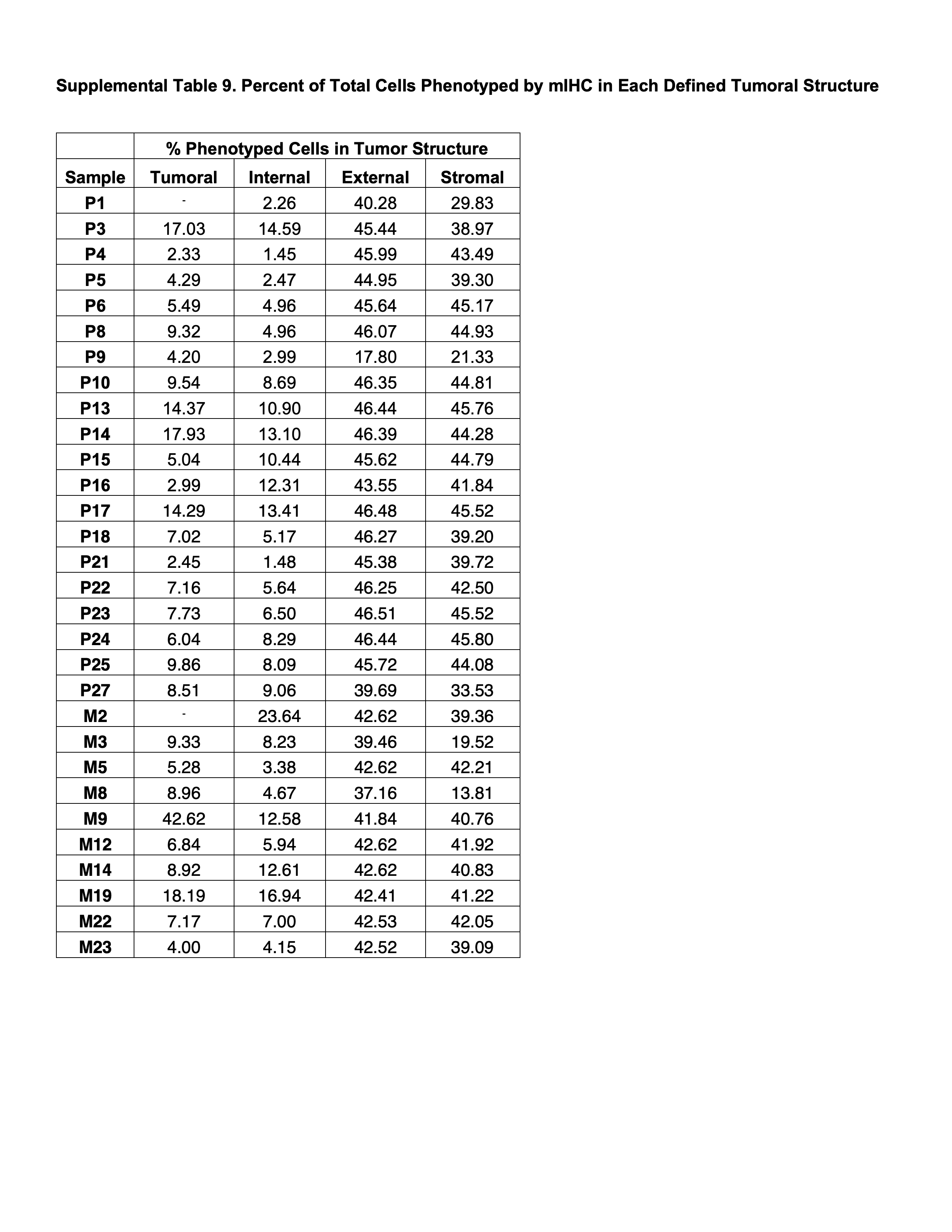

Supplement: Supplemental Table 9 — Percent of cells in each tumor structure [file can-25-1697_supplemental_table_9_suppst9.png]

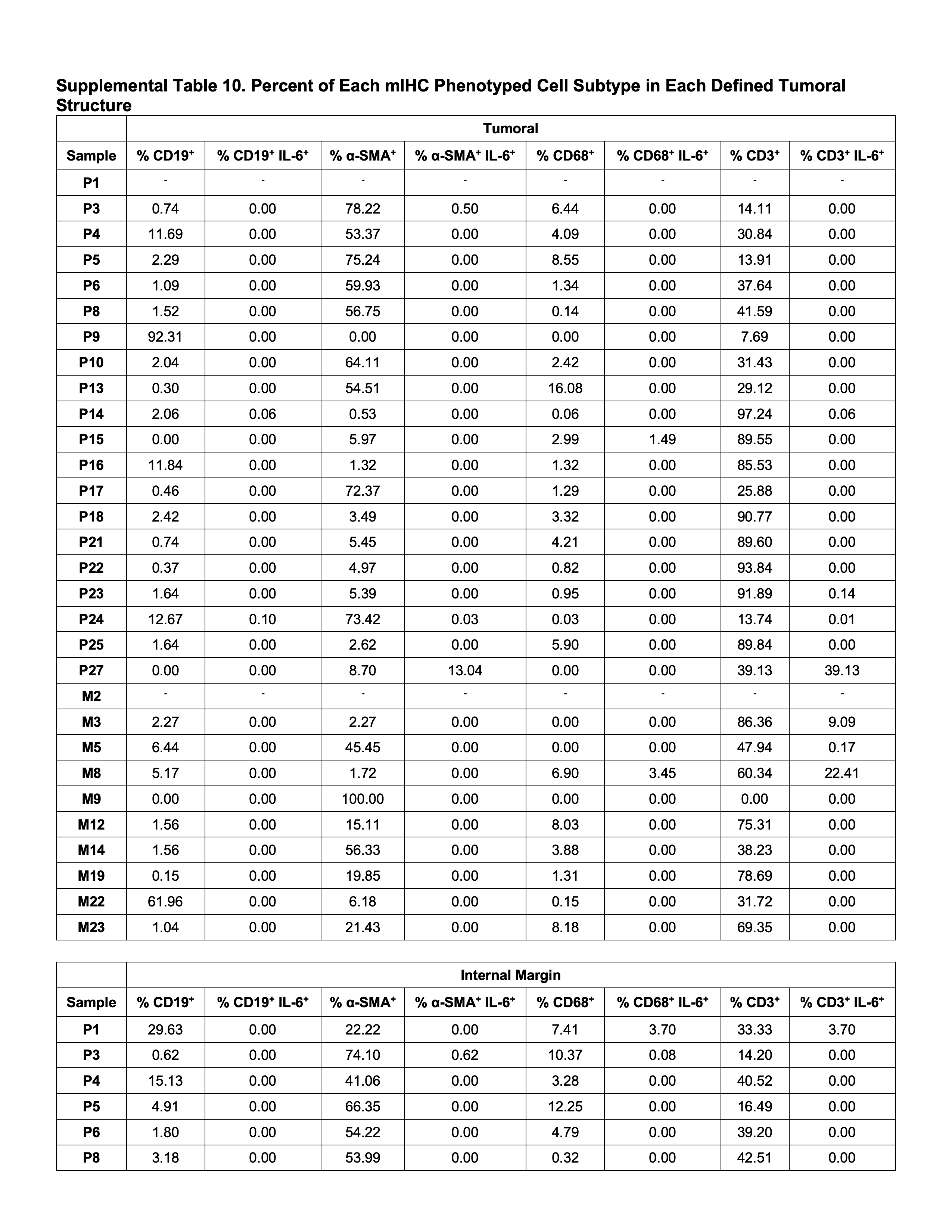

Supplement: Supplemental Table 10 — Percent phenotyped cell in tumor structure [file can-25-1697_supplemental_table_10_suppst10.png]

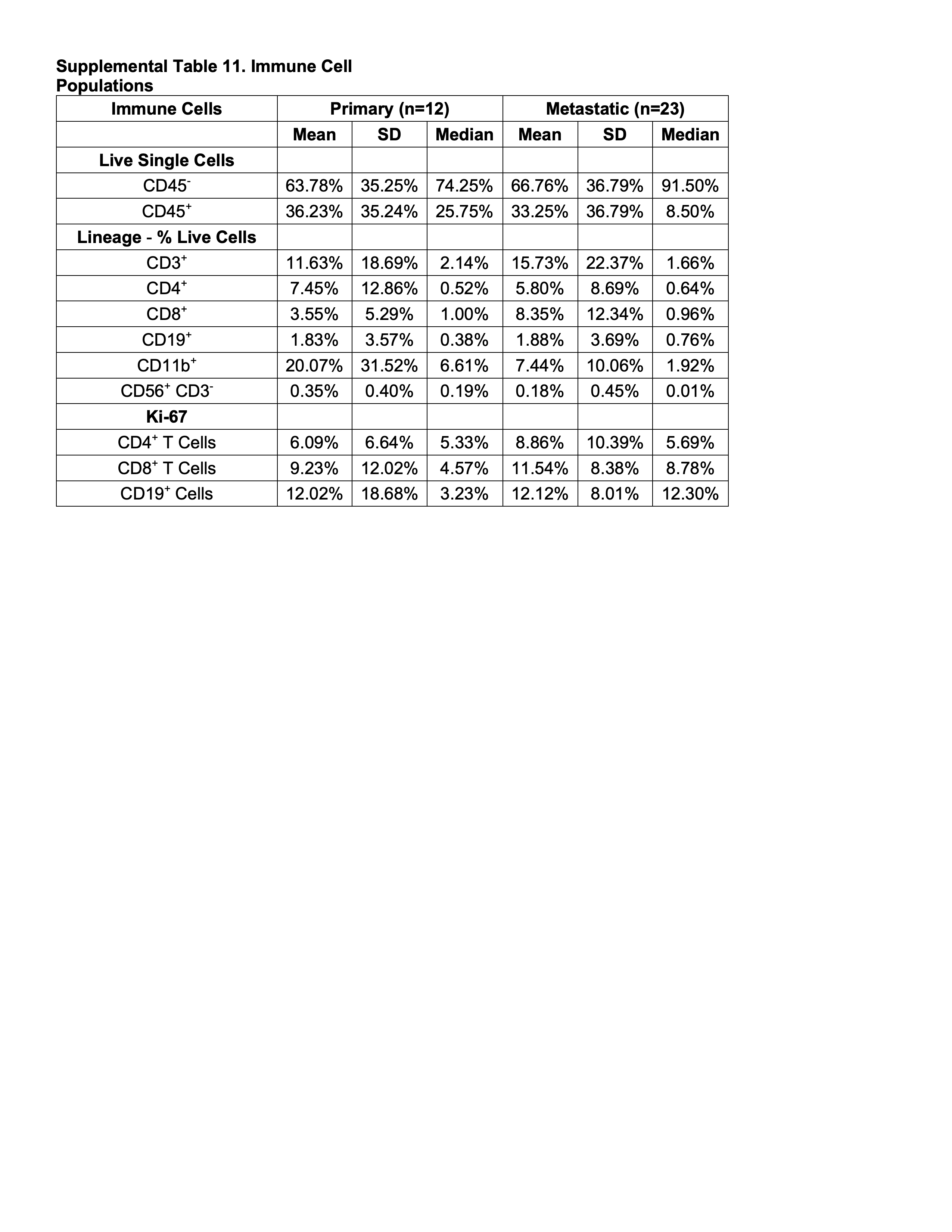

Supplement: Supplemental Table 11 — Immune cell populations [file can-25-1697_supplemental_table_11_suppst11.png]

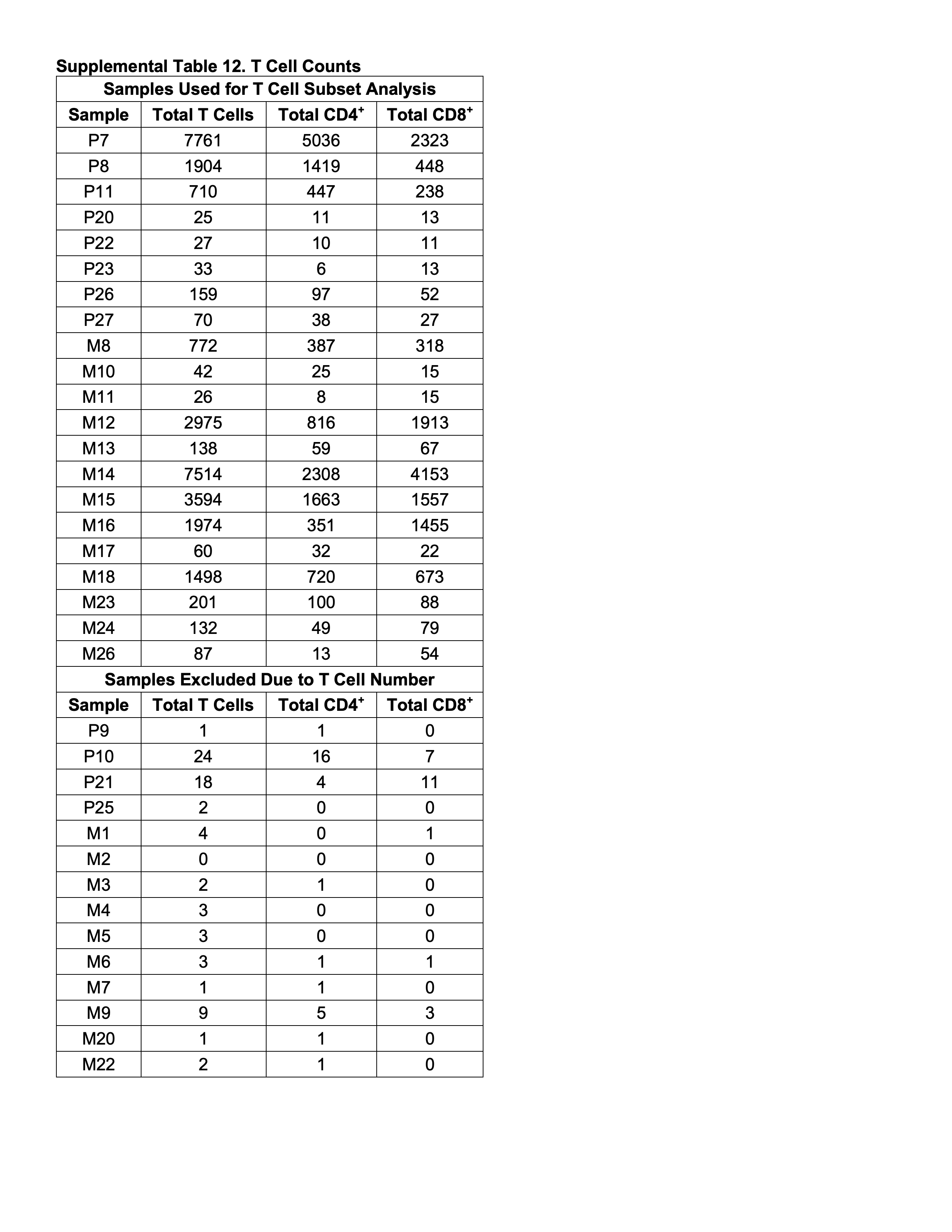

Supplement: Supplemental Table 12 — T cell counts [file can-25-1697_supplemental_table_12_suppst12.png]

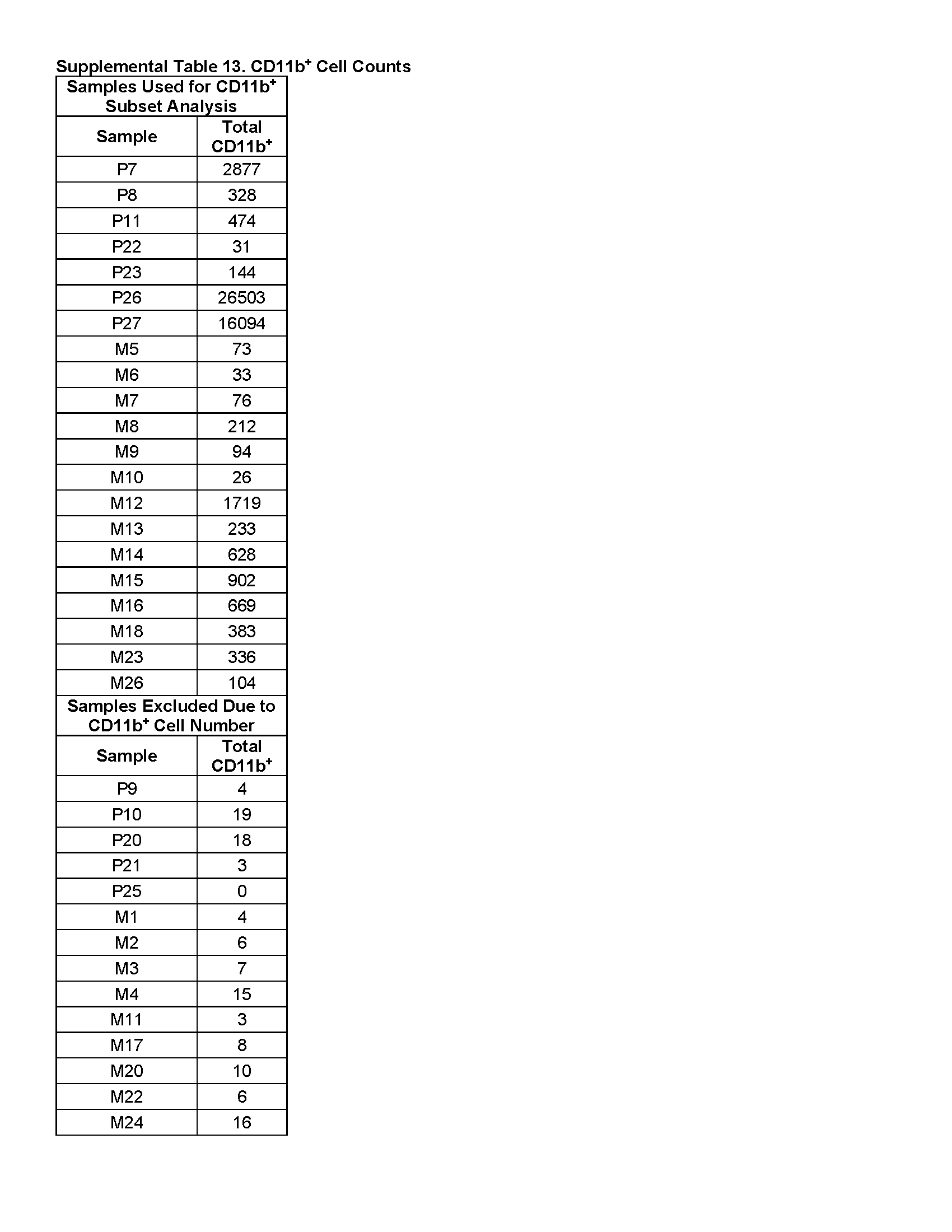

Supplement: Supplemental Table 13 — CD11b+ cell counts [file can-25-1697_supplemental_table_13_suppst13.png]

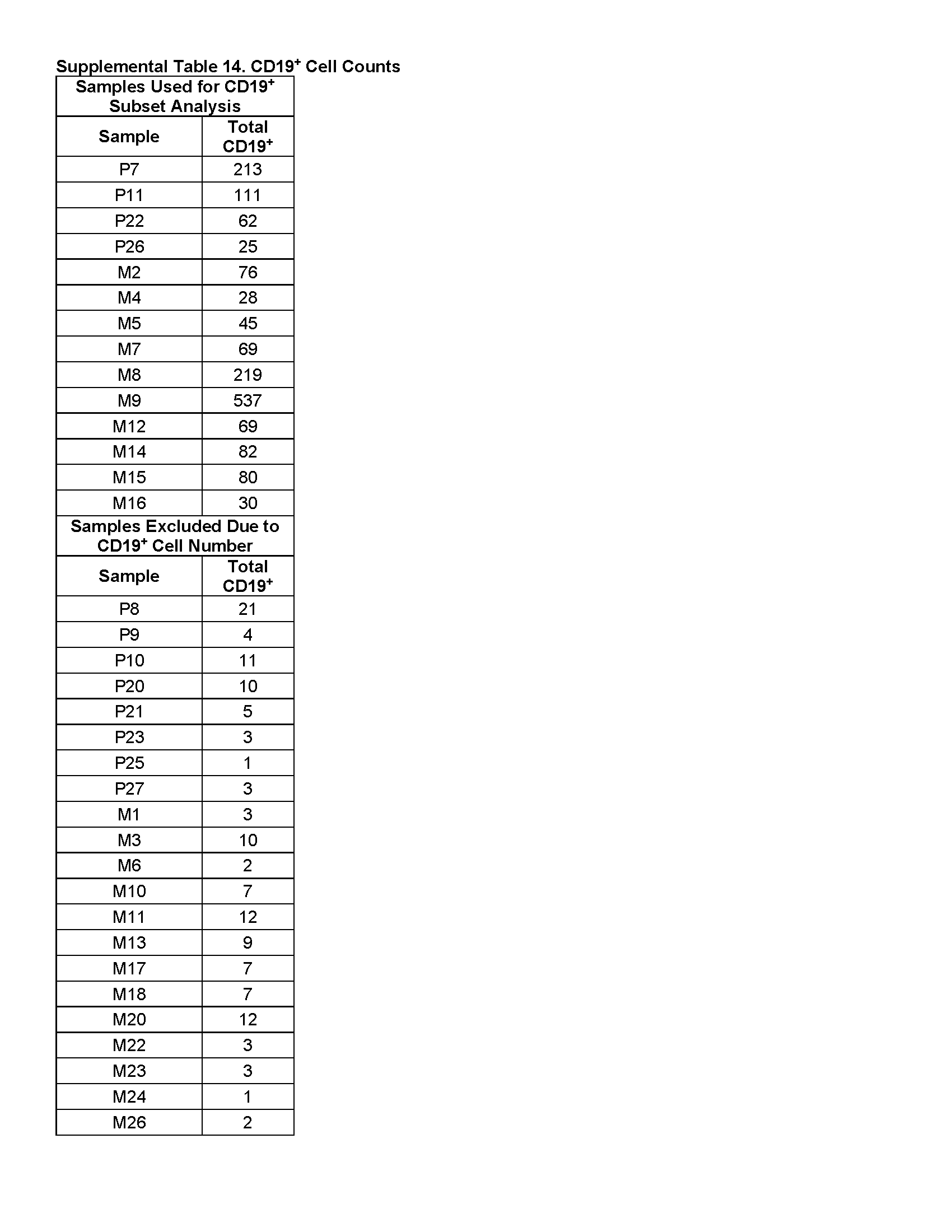

Supplement: Supplemental Table 14 — CD19+ cell counts [file can-25-1697_supplemental_table_14_suppst14.png]

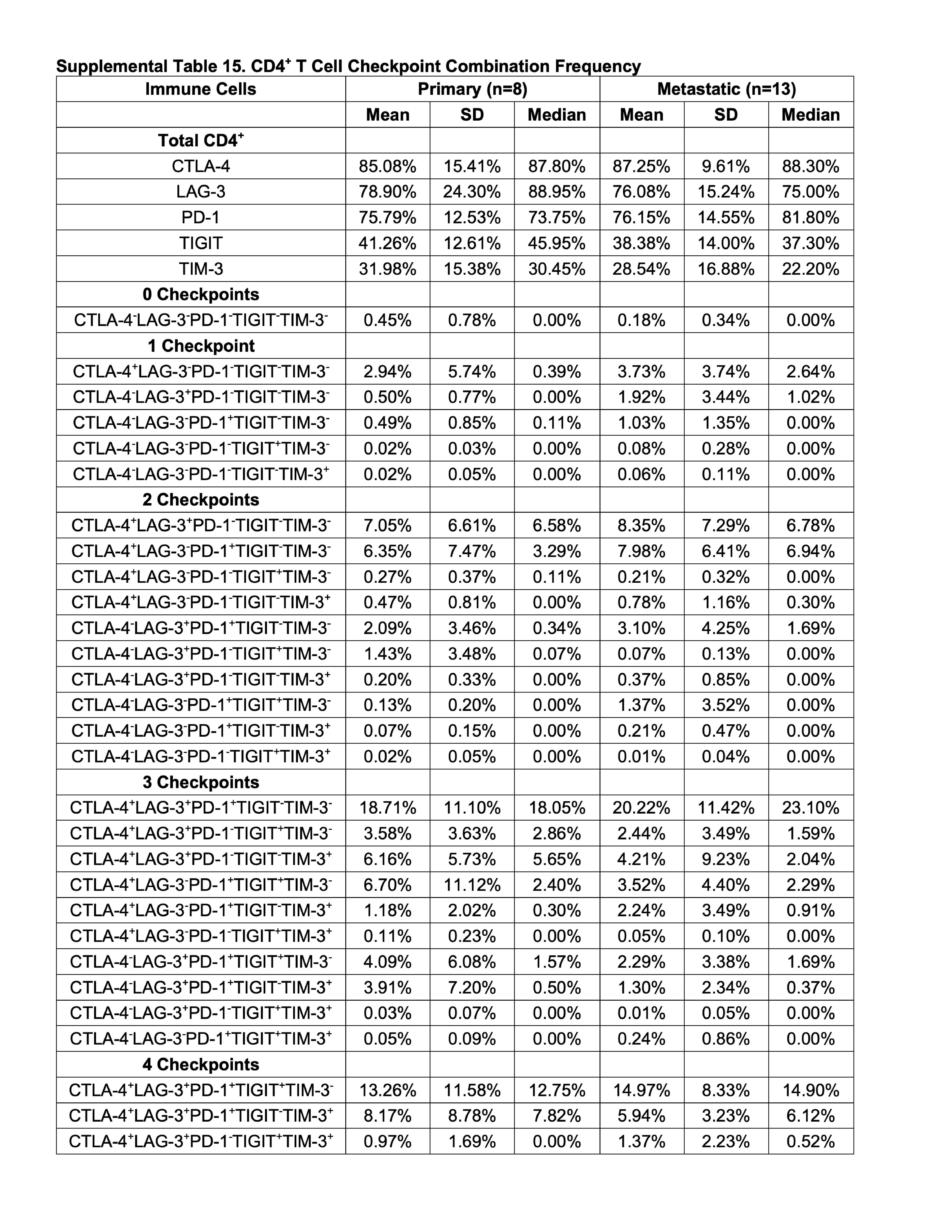

Supplement: Supplemental Table 15 — CD4+ checkpoints [file can-25-1697_supplemental_table_15_suppst15.png]

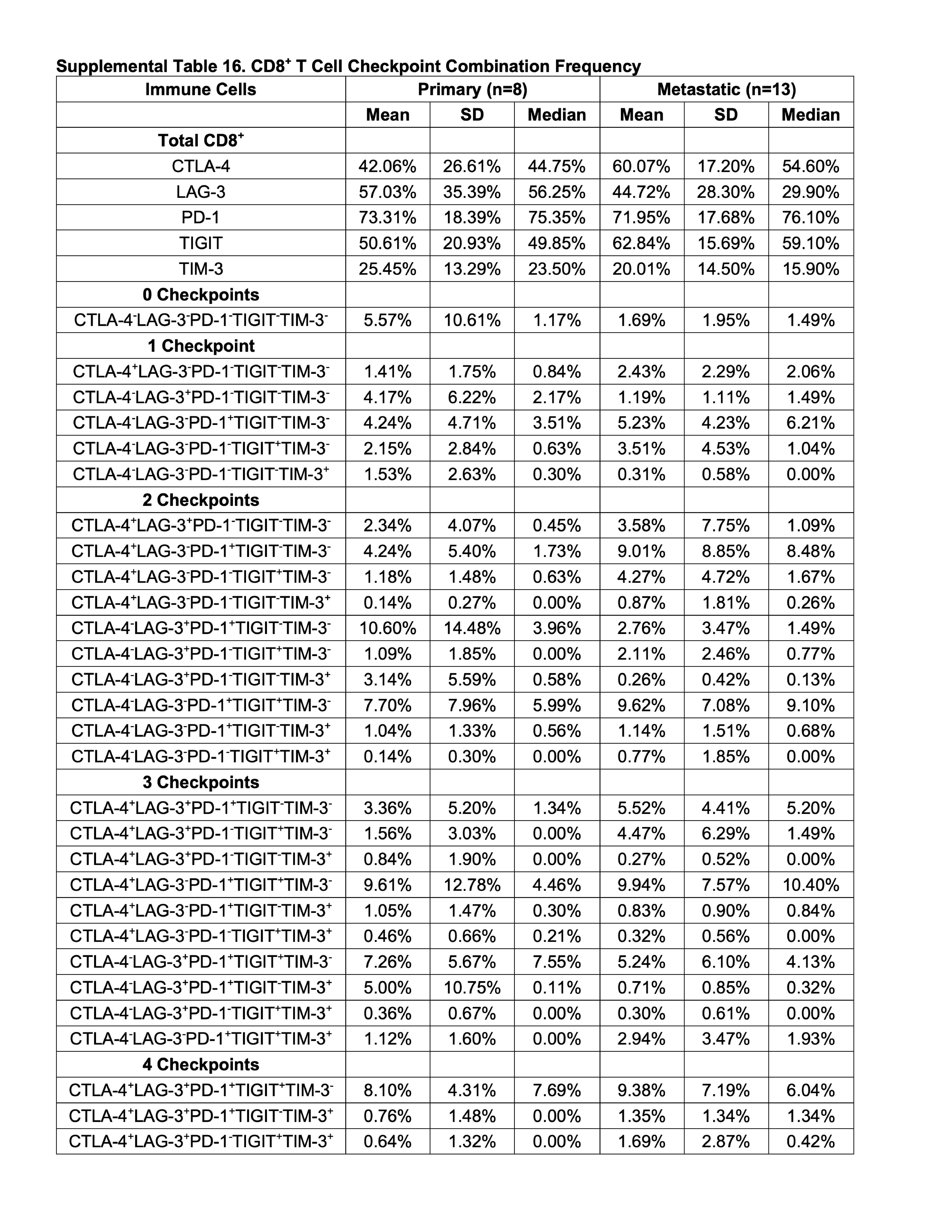

Supplement: Supplemental Table 16 — CD8+ checkpoints [file can-25-1697_supplemental_table_16_suppst16.png]
